# Nucleoporin Nup358 drives the differentiation of myeloid-biased multipotent progenitors by modulating HDAC3 nuclear translocation

**DOI:** 10.1126/sciadv.adn8963

**Published:** 2024-06-05

**Authors:** Valeria Guglielmi, Davina Lam, Maximiliano A. D’Angelo

**Affiliations:** ^1^ Cancer Metabolism and Microenvironment Program, NCI-designated Cancer Center, Sanford Burnham Prebys Medical Discovery Institute, La Jolla, CA, USA.; ^2^ Immunity and Pathogenesis Program, Infectious and Inflammatory Disease Center, Sanford Burnham Prebys Medical Discovery Institute, La Jolla, CA, USA.

## Abstract

Nucleoporins, the components of nuclear pore complexes (NPCs), can play cell type– and tissue-specific functions. Yet, the physiological roles and mechanisms of action for most NPC components have not yet been established. We report that Nup358, a nucleoporin linked to several myeloid disorders, is required for the developmental progression of early myeloid progenitors. We found that Nup358 ablation in mice results in the loss of myeloid-committed progenitors and mature myeloid cells and the accumulation of myeloid-primed multipotent progenitors (MPPs) in bone marrow. Accumulated MPPs in Nup358 knockout mice are greatly restricted to megakaryocyte/erythrocyte-biased MPP2, which fail to progress into committed myeloid progenitors. Mechanistically, we found that Nup358 is required for histone deacetylase 3 (HDAC3) nuclear import and function in MPP2 cells and established that this nucleoporin regulates HDAC3 nuclear translocation in a SUMOylation-independent manner. Our study identifies a critical function for Nup358 in myeloid-primed MPP2 differentiation and uncovers an unexpected role for NPCs in the early steps of myelopoiesis.

## Teaser

The nuclear pore complex component Nup358 regulates hematopoietic stem cells and is required for the development of blood cells.

## INTRODUCTION

Nuclear pore complexes (NPCs) are multiprotein channels that connect the nucleus to the cytoplasm and regulate the nucleocytoplasmic molecule exchange (*1*). NPCs are built by the repetition of ~30 different proteins known as nucleoporins. In recent years, NPCs have emerged as important regulators of tissue physiology, and nucleoporins have been found to play cell type– and tissue-specific roles (*2*, *3*). This helps to explain why mutations in different nucleoporins result in diseases with tissue-restricted phenotypes (*4*). Unfortunately, the physiological function of most individual nucleoporins has not yet been established. Nup358, also known as RanBP2, is the main component of the NPC cytoplasmic filaments (*5*, *6*). Nup358 shows high expression levels in bone marrow tissue (*7*, *8*), and alterations in this nucleoporin are associated with several blood disorders, in particular, myeloid malignancies (*4*). For example, chromosomal translocations involving *NUP358* gene with *FGFR1* lead to myelodysplastic syndrome/myeloproliferative neoplasms (MDS/MPNs) (*9*), while *NUP358* fusions with *ALK* are associated with acute myeloid leukemia (*10*, *11*). Nup358 is also misregulated in myelofibrosis and has been proposed as potential therapeutic target for this disease (*12*, *13*). Despite the clear link between Nup358 alterations and myeloid disorders, the role of this nucleoporin in physiological hematopoiesis has not been investigated.

During hematopoiesis, blood and immune cells are generated from hematopoietic stem cells (HSCs), a small pool of self-renewing and multipotent cells that reside in the bone marrow (*14*). HSCs give rise to multipotent progenitors (MPPs), a cell population that has lost self-renewal capacity but still retains multi-lineage potential. MPPs differentiate into lineage-restricted progenitors that generate mature immune cells of the innate and adaptive immune systems. The population of HSCs and MPPs are referred to as LSK (Lin^−^Sca1^+^cKit^+^) cells (*15*–*17*). Within the LSK population, priming toward specific lineages already occurs, and MPPs exist in a spectrum of different lineage-biased states. This includes megakaryocyte/erythrocyte primed (MPP2), granulocyte/macrophage primed (MPP3), and lymphoid primed (MPP4), which support lineage expansion and differentiation (*18*–*21*). The factors and mechanisms that regulate the priming of MPPs into different linages and the generation of lineage-committed progenitors are not entirely understood.

In this work, we identified that inactivation of Nup358 in mice strongly reduces the number of myeloid-committed progenitors and mature myeloid cells without affecting the amount of LSK cells. Detailed analysis of the LSK compartment revealed that Nup358 ablation decreases the number of short-term HSCs (HSC^ST^) and lymphoid-primed MPP4 but increases the number of megakaryocyte/erythrocyte-primed MPP2 cells. Functional studies established that the accumulation of MPP2 cells is due to their inability to differentiate and progress through the hematopoietic cascade. Mechanistically, we determined that Nup358 is required for proper function and nuclear translocation of the histone deacetylase HDAC3 and uncovered that these two proteins work together to regulate MPP2 cell cycle progression and differentiation. Our results uncover Nup358 as a key regulator of myeloid MPP homeostasis and provide the first insights into the role of NPCs in blood development.

## RESULTS

### Nup358 is required for myeloid cell development

To investigate whether Nup358 plays a role in hematopoiesis and avoid the embryonic lethality associated with its constitutive ablation in mice (*22*), we generated an inducible knockout line. *Nup358^fl/fl^* animals were crossed with *R26-CreER^T2^* (*CreER^T2^*) mice that constitutively express a tamoxifen-regulated Cre recombinase (fig. S1A). Tamoxifen treatment of adult *Nup358^fl/fl^CreER^T2^* mice (Nup358^−/−^ from now on) for three consecutive days efficiently ablated Nup358 in hematopoietic cells (fig. S1, B and C). Costaining with mAb414, which recognizes several nucleoporins (*23*), showed that Nup358 depletion does not result in major alterations of NPCs (fig. S1C). This is consistent with previous reports demonstrating that Nup358 is dispensable for NPC assembly (*24*).

**Fig. 1.**
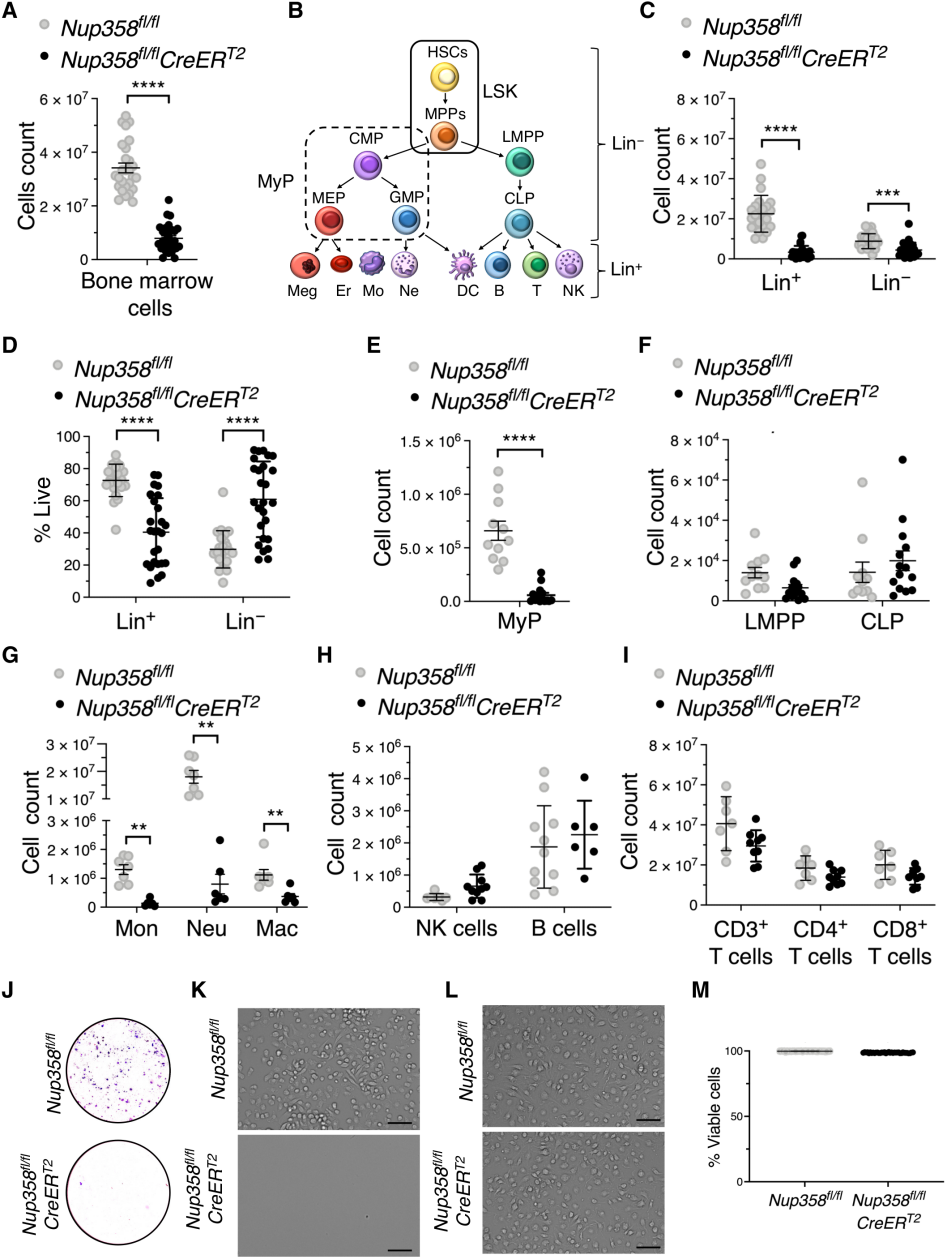
Nup358 is required for myeloid cell development. (**A**) Total bone marrow cells in *Nup358^fl/fl^* (control) and *Nup358^fl/fl^CreER^T2^* (Nup358 knockout) mice treated with tamoxifen. (**B**) Schematic illustration of hematopoiesis. HSCs, hematopoietic stem cells; MPPs, multipotent progenitors; LSK, Lin^−^Sca1^+^cKit^+^ cells; MyP, myeloid progenitors; CMP, common myeloid progenitors; MEP, megakaryocyte erythrocyte progenitors, GMP, granulocyte monocyte progenitors; LMPP, lymphoid-myeloid primed progenitors; CLP, common lymphoid progenitors; Meg, megakaryocytes; Er, erythrocytes; Mo, monocytes; Ne, neutrophils; DC, dendritic cells; B, B lymphocytes, T, T lymphocytes; NK, natural killer cells. (**C** to **H**) Flow cytometry analysis of bone marrow cells from control and Nup358 knockout mice. Mature immune cells (Lin^+^) and hematopoietic progenitors (Lin^−^) numbers (C), Lin^+^ and Lin^−^ percentage of viable cells (D). Number of MyP (E), LMPP and CLP (F). Number of monocytes (Mon), neutrophils (Neu), and macrophages (Mac) (G), NK cells, and B cells (H). (**I**) Number of CD3^+^, CD4^+^, and CD8^+^ T lymphocytes in spleen of control and Nup358 knockout mice. (**J**) Colony formation assay of bone marrow cells from control and Nup358 knockout mice in conditions promoting myeloid differentiation. (**K**) Representative images of macrophages differentiated in vitro from bone marrow cells of tamoxifen-treated *Nup358^fl/fl^* and *Nup358^fl/fl^CreER^T2^* mice. Scale bars, 75 μm. (**L**) Representative images macrophages differentiated in vitro from bone marrow cells of *Nup358^fl/fl^* and *Nup358^fl/fl^CreER^T2^* mice treated with tamoxifen after differentiation. Scale bars, 75 μm. (**M**) Viability of differentiated macrophages from (L). Data are means ± SD **P ≤ 0.01, ***P ≤ 0.001, ****P ≤ 0.0001 by either unpaired Student’s *t* test [(A), (E), and (M)] or multiple unpaired Student’s *t* test with Holm-Sidak method to correct for multiple comparisons [(C) and (D) and (F) to (I)]. Data are pooled from eight (A), six [(C) and (D)], three [(E) and (F)], two [(G) to (I)], and four (I) independent experiments with at least *n* = 3 mice of each genotype.

Five days after tamoxifen injection, bone marrows from Nup358^−/−^ mice showed significantly reduced cellularity compared to controls (Fig. 1A). Further characterization of these cells using the Lineage (Lin) antibody cocktail, which distinguishes mature blood cells (Lin^+^) from hematopoietic stem and progenitor cells (Lin^−^) (Fig. 1B), exposed a reduction of both cell populations (Fig. 1C). The decrease in Lin^+^ cells was more pronounced than the reduction in Lin^−^ cells, so that the frequency of this population in Nup358 knockouts was increased by approximately threefold (Fig. 1D). Detailed analysis of Lin^−^ cells revealed that Nup358 loss results in a strong reduction of myeloid progenitors (MyP), which include common myeloid progenitors (CMP), megakaryocyte erythrocyte progenitors (MEP), and granulocyte monocyte progenitors (GMP), without affecting lymphoid-myeloid primed progenitors (LMPPs) or common lymphoid progenitors (CLP) (Fig. 1, E and F, and fig. S1D). Consistent with the lower number of committed MyPs, bone marrows of Nup358^−/−^ mice exhibit fewer mature monocytes, neutrophils, and macrophages (Fig. 1G). Mature lymphoid cells including bone marrow natural killers, B cells, and splenic T cell populations, were not changed in these animals (Fig. 1, H and I). These data suggest a role for Nup358 in myeloid development. To confirm this, we performed colony formation assays in conditions that promote myeloid differentiation (*25*). We found that bone marrow cells from tamoxifen-treated *Nup358^fl/fl^CreER^T2^* mice failed to form colonies in vitro (Fig. 1J). Nup358 ablation also prevented the differentiation of bone marrow progenitors into macrophages (Fig. 1K). However, when Nup358 ablation was performed after hematopoietic progenitors had differentiated into macrophages, no effect on their numbers or survival was observed (Fig. 1, L and M, and fig. S1, E and F). The requirement of Nup358 for the development, but not the survival of mature myeloid cells, suggests a role for this nucleoporin in the differentiation of MyPs.

#### Nup358 deletion increases the number of myeloid-primed MPP2

**Fig. 2.**
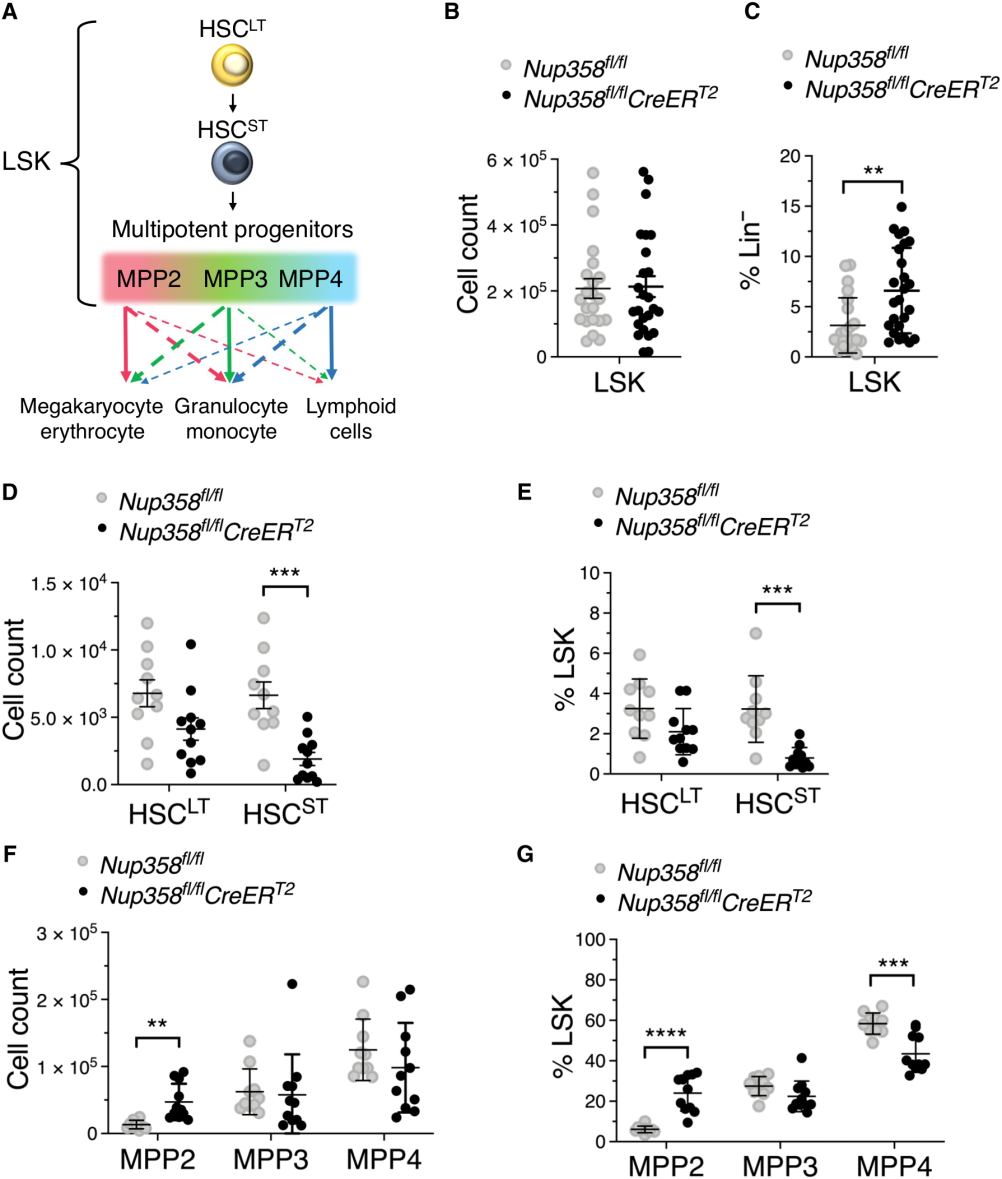
Nup358 knockouts have increased the number of megakaryocyte erythroid-primed MPP (MPP2). (**A**) Schematic illustration of the LSK subpopulations and MPP lineage priming. (**B**) Number of LSK cells in bone marrows of control and Nup358 knockout mice. (**C**) LSK cells as percentage of Lin^−^ cells. (**D**) Number of HSC^LT^ and HSC^ST^ in bone marrow of control and Nup358 knockout mice. (**E**) HSC^LT^ and HSC^ST^ as percentage of LSK cells. (**F**) Number of MPP2, MPP3, and MPP4 in bone marrows of control and Nup358 knockout mice. (**G**) Percentage of MPP2, MPP3, and MPP4 in the LSK population. Data are means ± SD. **P ≤ 0.01, ***P ≤ 0.001, ****P ≤ 0.0001 by either unpaired Student’s *t* test [(B) and (C)] or multiple unpaired Student’s *t* test with Holm-Sidak method to correct for multiple comparisons [(D) to (G)]. Data are pooled from six [(B) and (C)] and three [(D) to (G)] independent experiments with at least *n* = 3 mice of each genotype.

To investigate whether the reduced number of committed MyPs in Nup358^−/−^ mice is due to alterations in the early stages of hematopoiesis, we characterized the LSK cell compartment (Fig. 2A and fig. S2). We determined that Nup358 ablation did not affect the total number of LSK cells (Fig. 2B), although a significant increase in their frequency within the Lin^−^ progenitor population was observed in these mice due to the loss of committed MyPs (Fig. 2C). LSK cells constitute a heterogenous population that includes long-term HSCs (HSC^LT^), HSC^ST^, and MPPs (Fig. 2A). MPPs are further divided into subpopulations that are primed to generate different lineages (*18*–*21*). While MPP2 and MPP3 cells are skewed toward myeloid differentiation, being MPP2 primed for megakaryocyte/erythrocyte differentiation and MPP3 biased toward granulocyte/monocyte differentiation, MPP4 cells are primed toward lymphoid differentiation (Fig. 2A). Using a combination of previously described markers (Lin, Sca-1, cKit, CD135, CD48, and CD150) (fig. S2) (*20*), we determined that Nup358 ablation did not affect the HSC^LT^ population but strongly reduced the number and frequency of HSC^ST^ within the LSK population (Fig. 2, D and E). On the other hand, Nup358^−/−^ mice exhibited increased numbers and frequency of megakaryocyte/erythrocyte-primed MPP2 (Fig. 2, F and G). MPP3 and MPP4 numbers were not changed in Nup358^−/−^ animals, although the frequency of MPP4 within the LSK population was significantly reduced (Fig. 2, F and G). These findings show that Nup358 loss alters the distribution of the LSK cell population, reducing HSCs and increasing the number of myeloid-biased MPP2.

#### Nup358 function in myeloid differentiation is intrinsic to hematopoietic progenitor cells

**Fig. 3.**
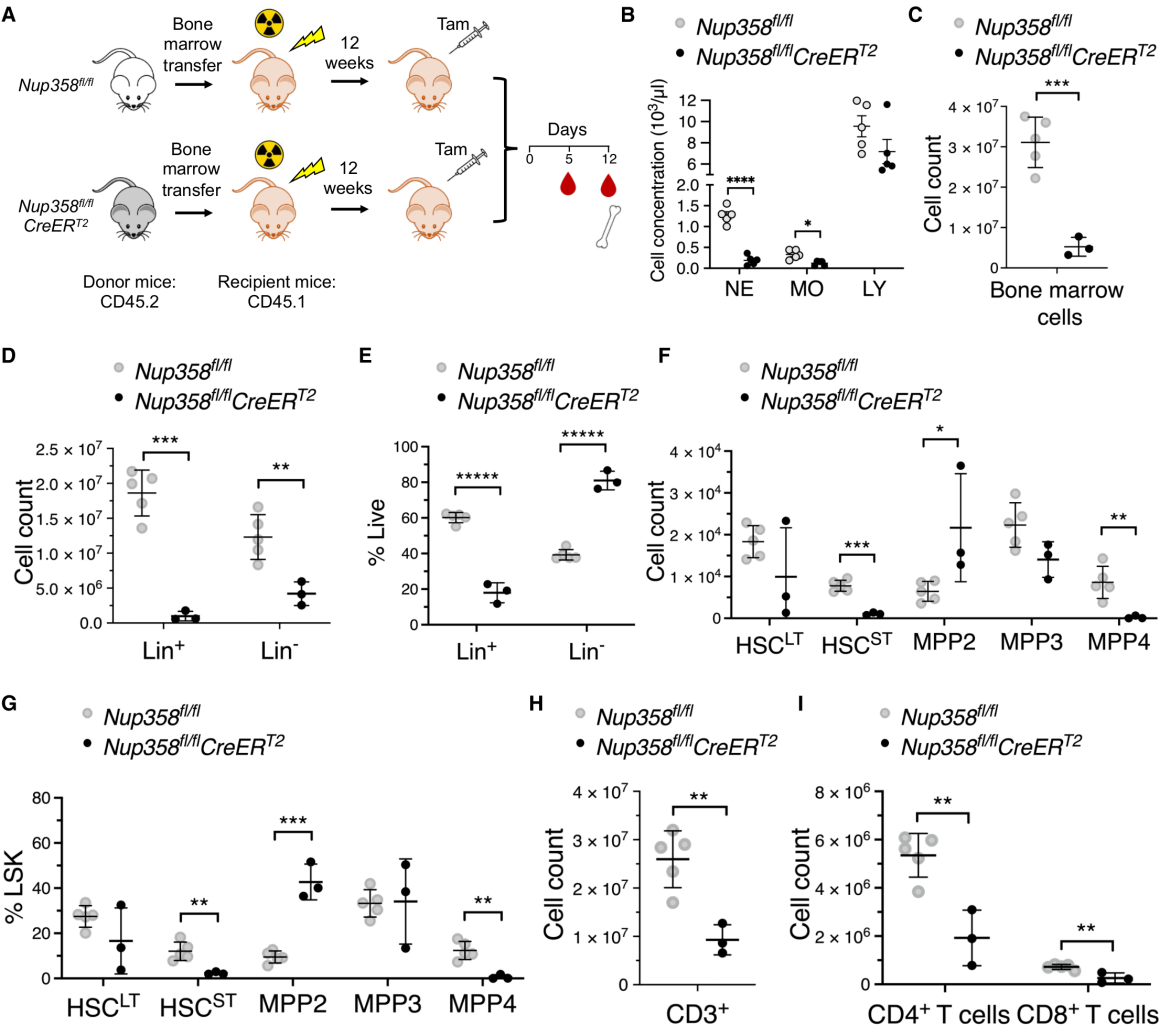
Nup358 role in myeloid development is intrinsic to hematopoietic progenitors. (**A**) Schematic illustration of the bone marrow transplantation experiments. (**B**) Number of neutrophils (NE), monocytes (MO), and lymphocytes (LY) per milliliter in peripheral blood of CD45.1 wild-type mice transplanted with bone marrow cells from either *Nup358^fl/fl^* or *Nup358^fl/fl^-CreER^T2^* mice after 12 weeks of reconstitution and 5 days after tamoxifen administration. (**C**) Total bone marrow cells from CD45.1 wild-type mice transplanted with bone marrow cells from either *Nup358^fl/fl^* or *Nup358^fl/fl^-CreER^T2^* mice at 12 days after tamoxifen administration. (**D** to **G**) Flow cytometry analysis on bone marrow cells isolated from CD45.1 wild-type mice transplanted with bone marrow cells from either *Nup358^fl/fl^* or *Nup358^fl/fl^-CreER^T2^* mice after 12 weeks of reconstitution and 12 days after tamoxifen administration. Lin^+^ and Lin^−^ cells as number (D) or percentage of viable bone marrow cells (E), and HSC^LT^, HSC^ST^ MPP2, MPP3, and MPP4 as number (F) or percentage of LSK cells (G) at 12 days after tamoxifen injection. (**H** and **I**) Flow cytometry analysis on splenocytes isolated from CD45.1 *wild-type* mice transplanted with bone marrow cells from either *Nup358^fl/fl^* or *Nup358^fl/fl^-CreER^T2^* mice after 12 weeks of reconstitution and 12 days after tamoxifen administration. Total CD3^+^ T lymphocytes (H), CD4^+^ T lymphocytes, and CD8^+^ T lymphocytes (I). Data are means ± SD. *P ≤ 0.05, **P ≤ 0.01, ***P ≤ 0.001, ****P ≤ 0.0001, *****P ≤ 0.00001 by unpaired Student’s *t* test [(C) and (H)] or multiple unpaired Student’s *t* test with Holm-Sidak method to correct for multiple comparisons [(D) to (G) and (I)].

Tamoxifen treatment of the *Nup358^fl/fl^CreER^T2^* mice results in the ablation of Nup358 in all tissues. To investigate whether the abnormalities observed in Nup358^−/−^ are due to intrinsic defects in the function of hematopoietic progenitors, we performed bone marrow transplantation assays. *Wild-type* recipient mice expressing the CD45.1 congenic marker were transplanted with bone marrow cells obtained from *Nup358^fl/fl^* or *Nup358^fl/fl^CreER^T2^* mice expressing the CD45.2 marker. After 12 weeks of reconstitution, the mice were treated with tamoxifen to eliminate Nup358 in hematopoietic cells, and the composition of bone marrow and peripheral blood was analyzed (Fig. 3A). Five days after the first tamoxifen injection, peripheral blood obtained from recipient mice transplanted with *Nup358^fl/fl^CreER^T2^* bone marrow cells showed complete recombination of exon 2 (fig. S3A) and reduced numbers of peripheral monocytes and neutrophils (Fig. 3B). Lymphocytes were again not affected (Fig. 3B). This agrees with our findings in *Nup358^fl/fl^CreER^T2^* full knockout mice. By 12 days after injection, mice showed signs of severe anemia (*26*), including reduced red blood cells, lower hemoglobin levels, and a drastic drop in hematocrit levels (fig. S3B). Congenic marker expression at this time point confirmed the successful engraftment of donor CD45.2 bone marrow cells in the CD45.1 *wild-type* recipients (fig. S3C). Analyses of bone marrow composition showed that similarly to *Nup358^fl/fl^CreER^T2^* full knockout mice, *wild-type* mice reconstituted with *Nup358^fl/fl^CreER^T2^* cells had a remarkable reduction of total bone marrow cells, Lin^+^ cells, and Lin^−^ cells, with the decrease in Lin^+^ cells being again more severe than the reduction of Lin^−^ cells (Fig. 3, C to E). Mice transplanted with *Nup358^fl/fl^CreER^T2^* bone marrow cells also showed a decrease in HSC^ST^ and the accumulation of MPP2 confirming our findings with full knockout mice (Fig. 3, F and G). Notably, at 12 days after tamoxifen, mice transplanted with *Nup358^fl/fl^CreER^T2^* cells also exhibited fewer MPP4 cells, which was not observed in *Nup358^fl/fl^CreER^T2^* full knockout mice at 5 days after the first tamoxifen injection (Fig. 3, F and G). Unfortunately, *Nup358^fl/fl^CreER^T2^* full knockout mice developed severe intestinal alterations by day 7 after tamoxifen injection that prevent us from studying hematopoietic phenotypes beyond 5 to 6 days of knockout. Consistent with decreased numbers of MPP4 cells, *wild-type* mice carrying *Nup358^fl/fl^CreER^T2^* hematopoietic cells also exhibited reduced numbers of lymphocytes in spleen and blood (Fig. 3, H and I). Together, these findings indicate that Nup358 function is intrinsic to hematopoietic progenitors and that its ablation results in the accumulation of the myeloid-primed MPP2, at the expense of HSCs and loss of the lymphoid-primed MPP4.

### Single-cell sequencing identified cell cycle and DNA replication as the most represented altered pathways in Nup358^−/−^ myeloid-primed MPP2

**Fig. 4.**
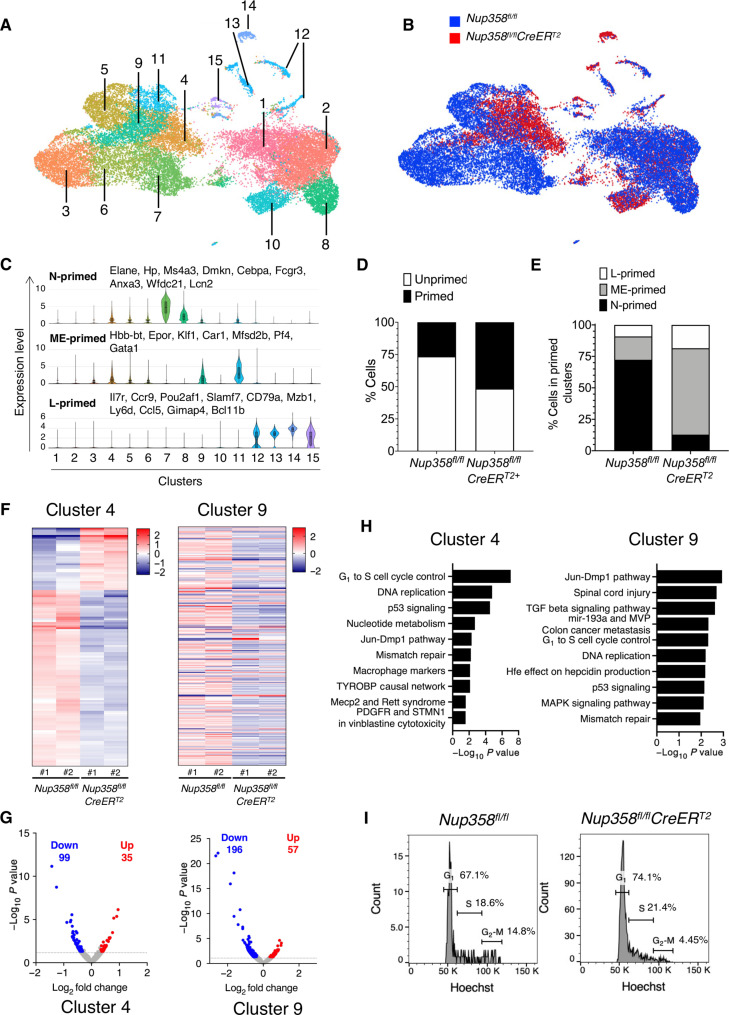
scRNA-seq analysis of Nup358^−/−^ LSK cells. (**A**) Uniform Manifold Approximation and Projection plot of control and Nup358 knockout cells showing 15 clusters identified by dimensionality reduction and unsupervised clustering. (**B**) Distribution of control (blue) and Nup358 knockout (red) cells among clusters. (**C**) Violin plots representing the aggregated expression of genes associated with neutrophils, megakaryocytes/erythrocytes, and lymphocytes in the identified clusters. (**D**) Distribution of control and Nup358 knockout cells in unprimed and primed clusters. (**E**) Distribution of control and Nup358 knockout cells in neutrophil-primed (N-primed), megakaryocytes/erythrocyte-primed (ME-primed), and lymphocyte-primed (L-primed) clusters. (**F**) Heatmaps showing genes differentially expressed between control and Nup358 knockout LSK cells from clusters 4 and 9 (fold change > ±1.25; *P* < 0.05). (**G**) Volcano plot of single-cell transcriptome data showing the differentially expressed genes in Nup358 knockout cells from clusters 4 and 9. Genes are organized by log_2_ fold change and –log_10_ *P* value. Genes below the cutoff (dashed line, *P* < 0.05) are shown in gray. (**H**) Ten most significantly altered pathways in Nup358 knockout LSK cells from cluster 4 and cluster 9 ranked by *P* value. (**I**) Cell cycle analysis on MPP2 in control (*Nup358^fl/fl^*) and Nup358 knockout (*Nup358^fl/fl^CreER^T2^*) by flow cytometry.

The decreased number of MPP4 explains the loss of circulating lymphocytes in mice reconstituted with Nup358 knockout cells. On the other hand, despite having increased numbers of the myeloid-primed progenitors MPP2, Nup358 knockout mice still fail to produce committed MyPs and mature myeloid cells. This suggests a role for Nup358 in regulating the differentiation of MPP2 cells. To determine how Nup358 might regulate the activity of MPP2 cells, we performed single-cell RNA sequencing (scRNA-seq) on LSK cells. This experiment was performed in duplicate with two control and two Nup358^−/−^ LSK samples, each prepared from a pool of three bone marrows isolated from individual animals (total of six animals per genotype) (fig. S4A). A total of 24,590 transcriptional profiles of LSK cells (15,516 from control and 9074 from Nup358^−/−^ cells) were sequenced to a median depth of approximately 50,000 reads per cell (table S1). Sequencing data were processed using Loupe software and the Rosalind single-cell sequencing analysis platform to generate cell clusters and identities. Through dimensionality reduction and unsupervised clustering with Uniform Mainfold Approximation and Projection, we distinguished a total of 15 clusters and changes in cell distribution between control and Nup358^−/−^ mice (Fig. 4, A and B, and table S2). Clusters were classified on the basis of the expression of lineage-associated genes and genes previously associated with different hematopoietic cell populations and cellular states (table S3) (*19*, *20*, *27*–*29*). We also examined cluster-associated genes using multiple algorithms including gProfiles, Metascape, BioGPS, and David Gene Ontology. Five clusters (1 to 3, 5, and 6) lacked expression of lineage-specific genes and were defined as stem/unprimed (fig. S4B). Nine clusters (4, 7 to 9, and 11 to 15) were considered lineage-primed because of the expression of lineage-specific genes (Fig. 4C and fig. S4B). These clusters were biased toward myeloid differentiation (neutrophil: clusters 7 and 8; MegE: clusters 4 and 9) or lymphoid differentiation (clusters 12 to 15) (Fig. 4C and fig. S4B). Neutrophil-primed clusters (N-primed) were characterized by up-regulation of markers such as Elane, Hp, Cebpa, and Anxa3 (Fig. 4C). MegE-primed clusters (ME-primed) were enriched in transcripts related to megakaryocyte and erythrocyte functions such as Hbb-bt, Epor, Klf1, and Gata1 (Fig. 4C). Lymphoid hallmark genes such as Il7r, Ccr9, and CD79a were increased in lymphoid-biased clusters (L-primed) (Fig. 4C). Clusters 10 and 11 were more difficult to classify because of a low number of genes selectively restricted to them. The two most significant markers for cluster 11, Car1 and Car2, are associated with MegE progenitors, suggesting that they are myeloid primed (table 3). Cluster 10 could not be classified and was not considered for subsequent analyses. Our scRNA-seq data corroborate the existence of lineage-biased MPPs and are in agreement with previous studies showing that the major LSK subpopulations defined with cell surface markers are more difficult to separate at the transcriptome level due to the heterogeneity of their transcriptional profiles (*18*–*21*, *28*–*30*).

Consistent with phenotypic analyses of bone marrow populations showing a decrease in HSC^ST^ and neutrophils and an increase in MPP2 cells (Figs. 1G and 2, D to G), scRNA-seq revealed that Nup358^−/−^ LSKs showed reduced numbers of cells in all stem/unprimed and neutrophil-primed clusters and a large expansion of the MegE-primed progenitors, clusters 4 and 9 (Fig. 4, D and E, and fig. S4, C to E). The expanded MegE-primed clusters showed expression of genes previously described as MPP2 markers (fig. S4D), suggesting that these clusters represent the phenotypically defined population MPP2. To investigate how Nup358 might regulate the early stages of myeloid differentiation, we characterized the transcriptional alterations of MPP2 clusters 4 and 9. A median of ~1700 genes per cell were detected in our single-cell sequencing studies. Cluster 4 showed 134 differentially expressed genes with control cells (Fold change >1.25; *P* < 0.05). Thirty-five genes were up-regulated, and 99 were down-regulated in Nup358^−/−^ (Fig. 4, F and G, and table S4). Cluster 9 had 253 differentially expressed genes, 57 up-regulated, and 196 down-regulated (Fig. 4, F and G, and table S4). Pathway analysis of deregulated genes showed alterations in cell cycle progression, DNA replication, and DNA damage response in both clusters (Fig. 4H). Differentially expressed genes within the cell cycle and DNA replication pathways are shown in fig. S4F. These findings suggest that Nup358 depletion might affect the ability of MPP2 cells to progress through the cell cycle. To investigate this, we analyzed the cell cycle distribution of the MPP2 population of control and Nup358^−/−^ mice by flow cytometry. Consistent with cell cycle alterations, we found that Nup358^−/−^ MPP2 cells accumulate in G_1_ and S phases (Fig. 4I).

### Nup358 regulates the function and nuclear translocation of HDAC3

**Fig. 5.**
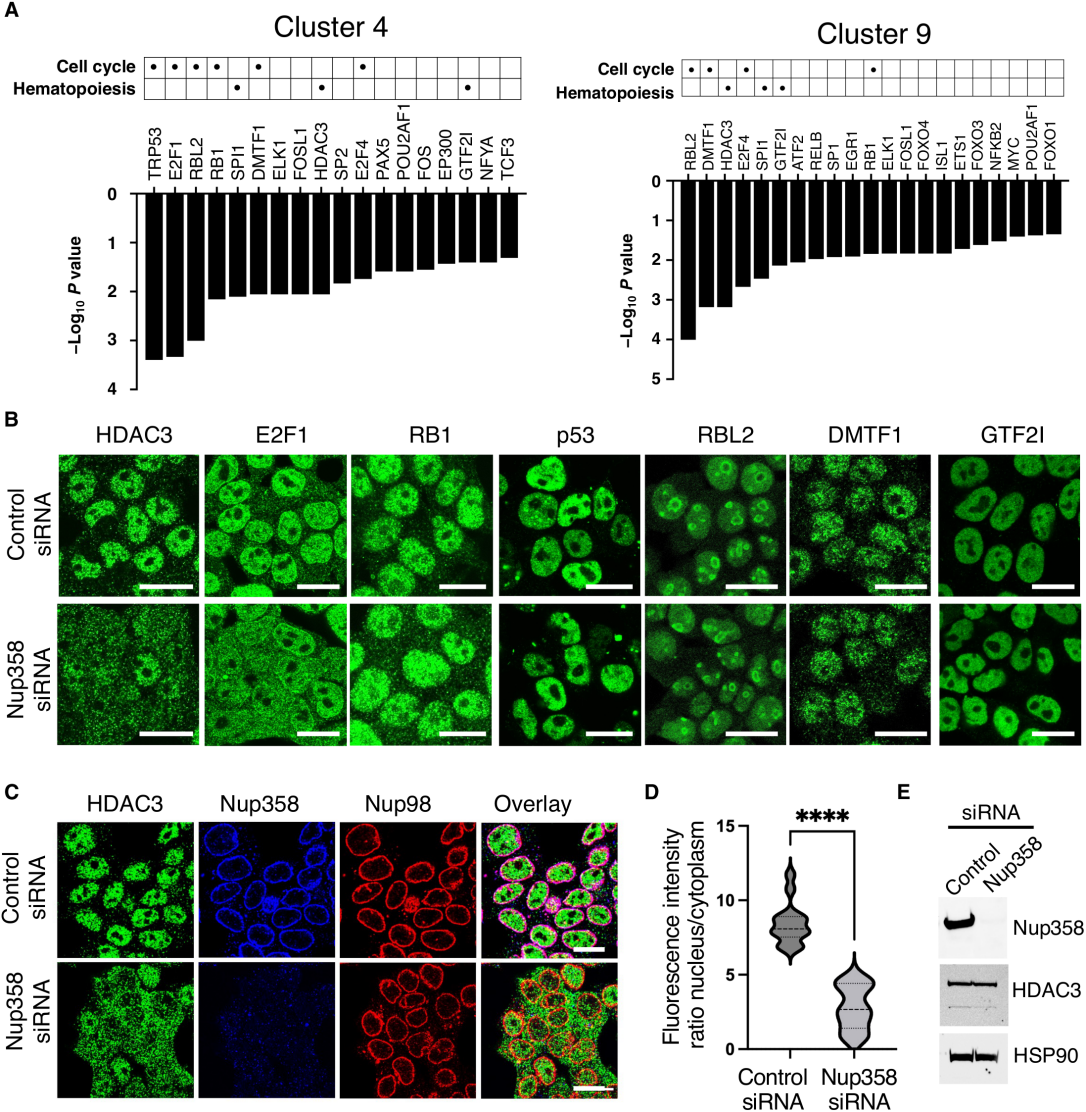
Nup358 is required for HDAC3 function and nuclear translocation. (**A**) Upstream transcriptional regulators (*P* < 0.05) predicted to be altered in Nup358 knockout LSK cells belonging to cluster 4 and cluster 9 ranked by *P* value. (**B**) The intracellular localization of HDAC3, E2F1, RB1, p53, RBL2, DMTF1, and GTF2I was analyzed by immunofluorescence in 293T cells transfected with control or Nup358-specific siRNAs. Scale bars, 25 μm. (**C**) HDAC3 intracellular localization was analyzed by immunofluorescence in 293T cells transfected with control or Nup358-specific siRNAs. Cells were costained with antibodies against nucleoporins Nup358 and Nup98. Scale bars, 25 μm. (**D**) Quantification of HDAC3 nuclear/cytoplasmic signal ratio in control or Nup358 knockdown cells. Data are expressed as mean ± SD. ****P ≤ 0.0001. (**E**) Protein levels of HDAC3 and Nup358 in cells transfected with scramble control or Nup358-specific siRNAs were analyzed by Western blot. HSP90 was used as loading control.

To investigate whether Nup358 might regulate MPP2 homeostasis by modulating the activity of specific transcriptional regulators, we analyzed the scRNA-seq data for altered upstream transcriptional regulators in Nup358 knockout MPP2 cells (clusters 4 and 9). This analysis revealed strong similarities between the two MPP2 clusters with an enrichment of cell cycle regulators including retinoblastoma (RB1), retinoblastoma-like protein 2 (RBL2), E2 factors (E2F1 and E2F4), tumor protein p53 (p53), and cyclin D–binding Myb-like transcription factor 1 (DMTF1) (Fig. 5A). Known regulators of hematopoiesis, including Salmonella pathogenicity island 1 (SPI1/PU.1), a key player of myeloid and lymphoid development (*31*–*35*); general transcription factor IIi (GTF2I), recently identified to have a function in erythro/megakaryopoiesis (*36*); and the histone deacetylase 3 (HDAC3), which has been shown to regulate the activity of MPPs (*37*), were also altered in Nup358^−/−^ MPP2 clusters. Considering that these factors shuttle between the nucleus and the cytoplasm and Nup358 main function in regulating the nuclear import of specific proteins (*38*), we investigated whether Nup358 ablation affected their nuclear translocation. For this, Nup358 was down-regulated in 293T cells using specific small interfering RNAs (siRNAs) and the subcellular localization of the most affected factors (RB1, RBL2, E2F1, DMTF1, p53, GTF2I, and HDAC3) was analyzed. Because SPI1/PU.1 is not expressed in 293T cells, its localization was analyzed in control and Nup358^−/−^ bone marrow–derived macrophages. We found that depletion of Nup358 strongly blocked the nuclear accumulation of HDAC3 (Fig. 5, B to E, and fig. S5, A to G). This was also observed in Nup358-depleted MOLM13 human acute myeloid leukemia cells (fig. S6A), confirming the requirement of this nucleoporin for the nuclear translocation of HDAC3 in hematopoietic cells. The nuclear accumulation of E2F1 was also affected by Nup358 depletion but to lesser extent than HDAC3 (Fig. 5B and fig. S5A). Total E2F1 protein levels were also decreased in Nup358-depleted cells (fig. S6B). Notably, a previous study identified that inhibition of HDAC3 activity reduces both E2F1 nuclear accumulation and total protein levels (*39*), suggesting that the alterations of E2F1 observed upon Nup358 loss could be a consequence of HDAC3 abnormalities. Consistent with this idea and phenocopying Nup358 down-regulation, we determined that selective inhibition of HDAC3 with RGFP966 (*40*) in these cells also reduced total E2F1 protein levels and E2F1 nuclear localization (fig. S6, B to D) but had no effect on RB1 localization, (fig. S6, E and F). In contrast to HDAC3, the localization of two other members of the class I HDAC family, HDAC1 and HDAC2, was not affected by Nup358 down-regulation (fig. S6, G and H). Together, these findings indicate that Nup358 selectively regulates HDAC3 nuclear import and support the idea that the E2F1 alterations observed in Nup358-depleted cells are likely a consequence of altered HDAC3 activity.

### Nup358 modulation of HDAC3 nuclear import is SUMOylation independent

**Fig. 6.**
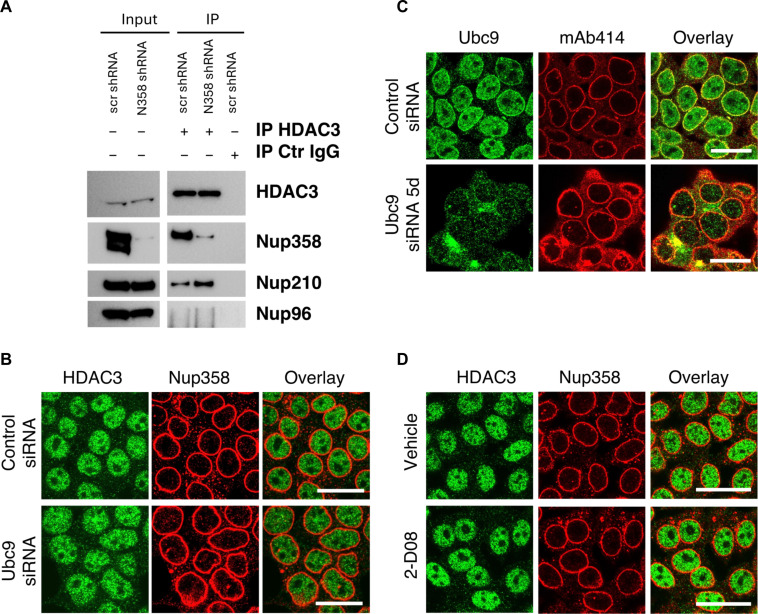
Nup358 regulates HDAC3 nuclear import in a SUMOylation-independent manner. (**A**) 293T cells were transduced with lentivirus carrying scramble control or Nup358-targeting shRNAs and treated with doxycycline to induce down-regulation of Nup358. HDAC3 was immunoprecipitated, and Nup358, Nup210, and Nup96 were analyzed by Western blot. (**B**) 293T cells were transfected with scramble control or Ubc9-specific siRNAs, and the localization of HDAC3 was analyzed after 120 hours by immunofluorescence. Cells were coimmunostained with an antibody against nucleoporin Nup358. Scale bars, 25 μm. (**C**) Ubc9 levels in 293T cells treated with control or Ubc9-specific siRNAs were analyzed by immunofluorescence 120 hours after transfection. Cells were coimmunostained with mAb414 antibody against NPCs. Scale bars, 25 μm. (**D**) 293T cells were treated for 72 hours with the selective SUMOylation inhibitor 2-D08 (25 μM), and the localization of HDAC3 was analyzed by immunofluorescence. Cells were costained with an antibody against nucleoporin Nup358. Scale bars, 25 μm.

Nup358 has SUMO E3 ligase activity and works together with Ubc9 and RanGAP1-SUMO1 to SUMOylate many cellular targets (*41*, *42*). A previous study identified that the interaction between HDAC4 and Nup358 is required for HDAC4 SUMOylation and recruitment to target genes (*43*). Coimmunoprecipitation assays in protein extracts from control or Nup358-depleted cells confirmed that Nup358 also interacts with HDAC3 (Fig. 6A). Notably, HDAC3 also immunoprecipitated Nup210 but not Nup96. However, we found that depletion of Nup210 had no effect on HDAC3 nuclear localization (fig. S7A). This indicates that HDAC3 can associate with specific NPC components and confirms a selective role for Nup358 in regulating its nuclear accumulation. To investigate whether HDAC3 nuclear translocation is regulated by Nup358-mediated SUMOylation, we immunoprecipitated HDAC3 from control and Nup358-depleted cells and analyzed the presence of SUMO modifications by Western blot or mass spectrometry. Although the proteomic analyses confirmed the interaction between Nup358 and HDAC3 (table S5), both of these approaches failed to detect SUMO peptides on HDAC3. This is consistent with previous findings where the Ubc9/Nup358 complex was found to modify several HDACs but not HDAC3 (*43*). Considering that SUMOylation can be difficult to detect, to further exclude its involvement in the nuclear translocation of HDAC3, we inhibited SUMOylation in 293T cells by either down-regulating Ubc9, the only cellular SUMO E2 ligase (*44*), or by pharmacologically inhibiting the transfer of the SUMO peptide to substrates with 2-D08 inhibitor (*45*), and analyzed HDAC3 localization by immunofluorescence. We found that despite both treatments being highly effective at reducing SUMOylation (fig. S7B), they did not affect HDAC3 nuclear accumulation (Fig. 6, B to D), confirming that HDAC3 nuclear localization does not require SUMOylation. Together, these data indicate that Nup358 regulates HDAC3 nuclear localization in a SUMOylation-independent way.

### Nup358 controls MPP2 homeostasis by regulating HDAC3 function

**Fig. 7.**
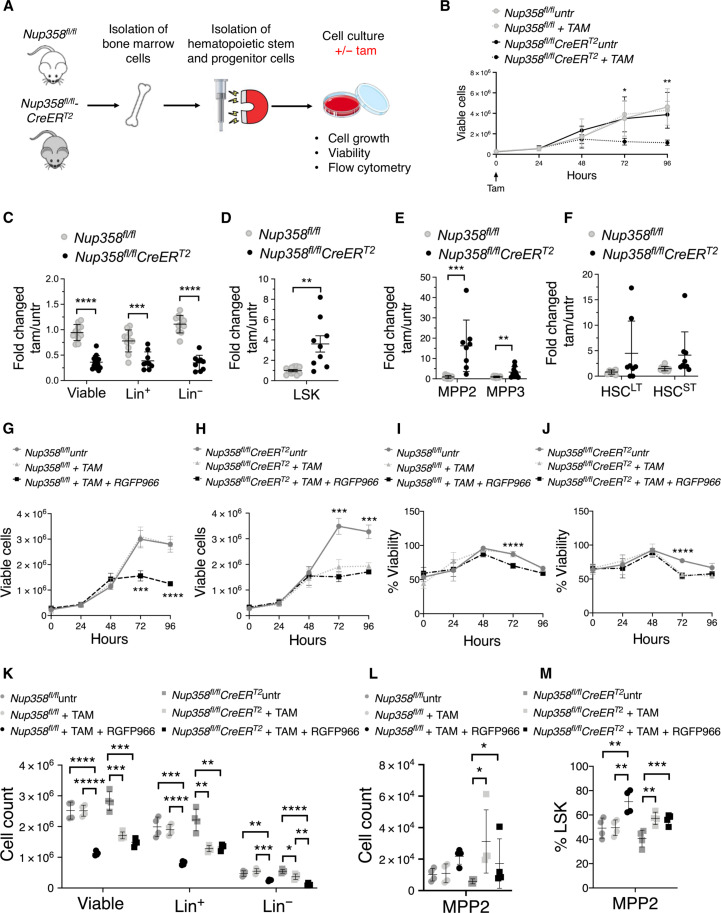
Nup358 and HDAC3 work together to regulate MPP2 homeostasis. (**A**) Schematic illustration of the in vitro myeloid differentiation of primary hematopoietic progenitors. (**B**) The total number of viable cells in cultures of *Nup358^fl/fl^* and *Nup358^fl/fl^CreER^T2^* hematopoietic progenitors treated with tamoxifen in vitro was measured over time. (**C** to **F**) Flow cytometry analysis of hematopoietic progenitor cultures 96 hours after treatment with tamoxifen. Fold change relative to vehicle-treated cells. (**G** to **J**) Cell growth and viability of *Nup358^fl/fl^* and *Nup358^fl/fl^CreER^T2^* hematopoietic progenitor cultures treated with either tamoxifen or vehicle for 96 hours and HDAC3 inhibitor RGFP966 or vehicle for 48 hours. (**K** to **M**) Flow cytometry analysis of hematopoietic progenitor cultures treated with either tamoxifen or vehicle for 96 hours and HDAC3 inhibitor RGFP966 or vehicle for 48 hours. Data are means ± SD *P ≤ 0.05, **P ≤ 0.01, ***P ≤ 0.001, ****P ≤ 0.0001, *****P ≤ 0.00001 by unpaired Student’s *t* test (D) or multiple unpaired Student’s *t* test with Holm-Sidak method to correct for multiple comparisons [(C), (E), and (F)] or analysis of variance (ANOVA) with Turkey correction for multiple comparison [(K) to (M)]. Data in (B) to (F) are representative of two independent experiments with at least *n* = 3 biological samples of each genotype.

Our findings indicate that Nup358 is required for the nuclear translocation of HDAC3. A previous study identified a critical function for HDAC3 in the regulation of MPP cells (*37*). Notably, deletion of HDAC3 in hematopoietic cells accurately phenocopies Nup358 knockout mice (*37*). HDAC3 knockout animals exhibit reduced bone marrow cellularity, lower numbers of Lin^+^ and Lin^−^ cells with increased the representation of the Lin^−^ progenitor/stem cell population, loss of MPP4 cells, and accumulation of myeloid-primed MPP, particularly the MegE-biased MPPs (or MPP2s) (*37*). In the same study, HDAC3 was found to be required for proper DNA replication and cell cycle progression in hematopoietic progenitor cells (*37*). These studies, together with our findings, suggest that Nup358 might regulate MPP2 homeostasis by modulating HDAC3 function. To investigate this hypothesis, we established an in vitro model system for myeloid differentiation (Fig. 7A). Lin^−^ progenitor cells were isolated from control (*Nup358^fl/fl^*) and *Nup358^fl/fl^CreER^T2^* mice and cultured in the presence of stem cell factor (SCF), interleukin-6 (IL-6), and IL-3 to promote their expansion and differentiation toward the myeloid lineage. Tamoxifen was added after 24 hours, and cultures were analyzed over time. Nup358 ablation was confirmed by polymerase chain reaction and immunofluorescence (fig. S8, A and B). Depletion of Nup358 in isolated progenitor cells significantly affected cell proliferation and culture expansion (Fig. 7B). Flow cytometry analyses 96 hours after tamoxifen treatment revealed a significant reduction in Lin^+^ and Lin^−^ cells and an increased number of LSK cells (Fig. 7, C and D, and fig. S8, C to F). The increase in LSK cells was primarily due to higher numbers of myeloid-primed MPP2 cells, although MPP3 cells were also increased in Nup358^−/−^ in vitro cultures (Fig. 7E and fig. S8E). These findings indicate that the in vitro differentiation model recapitulates our in vivo studies and further confirm that Nup358 plays an intrinsic role in the differentiation of hematopoietic progenitors.

To investigate the role of HDAC3 in Nup358 function using this model system, hematopoietic progenitors from control and *Nup358^fl/fl^CreER^T2^* mice were isolated and cultured in vitro in the presence of tamoxifen alone or in combination with the selective HDAC3 inhibitor RGFP966 (fig. S8G). Inhibiting HDAC3 in control cells reduced the number of proliferating cells in a similar way to Nup358 ablation (Fig. 7G). On the other hand, HDAC3 inhibition in Nup358-depleted cells did not further affect cell growth (Fig. 7H). The same was observed with cell viability (Fig. 7, I and J). The lack of synergism with the RGFP966 inhibitor indicates that Nup358 and HDAC3 work in the same genetic pathway. To determine whether HDAC3 inhibition also results in accumulation of MPP2, cell cultures were analyzed 48 hours after inhibition for Lin^+^, Lin^−^, LSK, and MPP cells. Consistent with the phenotype induced by HDAC3 deletion in mice (*37*), blocking HDAC3 activity in control cells decreased the numbers of viable, Lin^+^, and Lin^−^ cells but did not affect LSK cells (Fig. 7K and fig. S8H). Notably, HDAC3 inhibition in these cells also resulted in accumulation of the MPP2 (Fig. 7, L and M), mimicking the phenotypes of Nup358-depleted hematopoietic progenitor cultures and Nup358^−/−^ mice. Again, inhibiting HDAC3 activity in Nup358^−/−^ hematopoietic progenitors did not further enhance the increase of MPP2 cells observed with Nup358 ablation (Fig. 7, L and M). Currently, there are no specific inhibitors for other class I HDAC family members that allow us to study the effect of inhibiting each HDAC individually in our in vitro differentiation system. Nevertheless, we found that, differently from HDAC3 inhibition, treatment with the dual HDAC1/HDAC2 inhibitor Romidepsin results in the complete loss of LSK cells in both control and Nup358 knockout hematopoietic progenitors (fig. S9, A and B). These results further support the specificity of HDAC3 function in MPP differentiation and are consistent with previous genetic studies showing that single deletion of HDAC1 or HDAC2 does not affect hematopoietic progenitors, while the double knockout results in complete loss of LSK and progenitor cells. (*46*). Together, our findings indicate that Nup358 and HDAC3 work together to regulate the developmental progression of MPP2 progenitors.

## DISCUSSION

Evidence accumulated in recent years shows that the expression of nucleoporins can vary among cells and tissues and that these proteins can play cell type– and tissue-specific functions (*2*, *3*). However, the physiological functions and molecular mechanisms of action of most individual nucleoporins still remain unknown. In this work, we provide evidence that Nup358, a nucleoporin highly expressed in bone marrow and associated with myeloid disorders, regulates the early stages of myeloid development. We found that inactivation of Nup358 in mice leads to alterations within the population of early hematopoietic progenitors, reducing the number of HSCs and lymphoid-primed MPP4 cells and increasing the number of megakaryocyte/erythrocyte-primed MPP2 cells. As expected, the reduced number of MPP4 cells results in the loss of lymphoid-committed progenitors and mature lymphocytes. On the other hand, although the number of MPP2 cells is increased in Nup358 knockout mice, these cells fail to produce myeloid-committed progenitors and these animals show a notable reduction in mature myeloid cells. Our work uncovered a role for Nup358 in regulating the developmental progression of MPP2, revealing a previously unknown role for NPCs in myelopoiesis.

Mechanistically, we determined that Nup358 regulates MPP2 homeostasis by modulating HDAC3 function. Notably, a previous study identified HDAC3 as an important regulator of hematopoietic progenitor homeostasis (*37*), although the factors and mechanisms that regulate its functions remained unknown. Deletion of HDAC3 in the hematopoietic compartment were shown to lower the number of lymphoid-primed MPPs and peripheral lymphocytes and increased myeloid-primed MPPs with loss of bone marrow myeloid cells, which accurately phenocopies Nup358 knockout mice (*37*). In progenitor cells, HDAC3 was found to be required for DNA replication and cell cycle progression (*37*), providing an explanation for the alterations in DNA replication and cell cycle processes in Nup358 knockout MPP2 cells and for their accumulation in G_1_ and S phases. Our studies provide evidence that Nup358 interacts and selectively regulates the nuclear translocation of HDAC3. This supports a previous study showing that Nup358 is required for the nuclear translocation of specific cellular proteins (*38*) and provides additional confirmation that Nup358 is dispensable for general nuclear import (*47*–*49*). Nup358 has been found to regulate nuclear import through different mechanisms (*38*, *50*, *51*). It can act as a scaffold that helps to recruit import receptors to NPCs increasing the efficiency of nuclear translocation or facilitate import by directly interacting with selective cargos through different domains in a receptor-independent manner (*38*). Nup358 can also modulate nuclear import indirectly by SUMOylating target proteins (*43*). HDAC3 contains nuclear export and nuclear localization signals that mediate its active shuttling between the nucleus and the cytoplasm (*52*, *53*). The mechanism via which Nup358 controls HDAC3 nuclear import is currently unknown. However, considering that HDAC3 nuclear translocation does not require SUMOylation and that this protein interacts with Nup358, it is possible that this nucleoporin might directly enhance HDAC3 nuclear import or its association with import receptors. Future studies will be required to establish how Nup358 promotes HDAC3 translocation and to identify HDAC3 downstream targets in MPP2 cells.

It has been shown that during inflammation and blood loss, HSCs prioritize the generation of myeloid-biased MPPs to restore blood production, in a process referred to as emergency hematopoiesis (*20*, *54*). Besides increasing MPP2 numbers, Nup358 ablation also causes a decrease in HSC^ST^. This might represent a compensatory response aimed at restoring blood generation triggered by the loss of MyPs and mature myeloid cells. However, the MPP2 cells produced in these animals are unable to proceed through the myeloid differentiation program leading to compromised myelopoiesis. Consistent with the Nup358/HDAC3 axis regulating the developmental progression of megakaryocyte/erythrocyte-biased MPP2 and myelopoiesis, broad-spectrum HDAC inhibitors have been shown to affect the activity of HSCs and committed progenitors, particularly affecting the erythroid lineage. A study using a cell line–based differentiation model and transplanted fetal liver progenitor cells identified a role for HDAC1 and HDAC2 in erythrocyte differentiation (*55*, *56*). These enzymes were also found to reduce the number of mature erythrocytes when codepleted in vivo (*56*, *57*). Nevertheless, the stage of the megakaryocyte-erythrocyte developmental process at which HDAC1 and HDAC2 work and if their depletion affects the distribution or function of MPP populations has not been established. We found that knockout of Nup358 specifically impairs the nuclear import of HDAC3 but does not affect the localization of HDAC1 and HDAC2. While our results cannot discard a role for Nup358 in regulating the activity rather than the localization of other HDAC members, our findings showing that HDAC3 inhibition is sufficient to recapitulate the phenotypes of Nup358 knockouts, together with our scRNA-seq analyses demonstrating the selective alteration of HDAC3 transcriptional network in Nup358 knockout MPP2, support a critical role for the Nup358/HDAC3 in the early stages of myeloid development.

Nucleoporins have long been associated with blood disorders. The first connection between a nucleoporin and a blood cancer, Nup214 in leukemia, was described almost 30 years ago (*58*). After that, several other nucleoporins, including Nup358, have been linked to hematological malignancies (*4*, *9*–*11*, *59*, *60*). Yet, a role for nucleoporins in physiological hematopoiesis had not been described so far. Our discovery that Nup358 is required for the differentiation of myeloid-biased MPPs not only provides the first insights into how nucleoporins contribute to the regulation of blood development but could help to establish how alterations in Nup358 function contribute to myeloid malignancies. Chromosomal translocations involving the *NUP358* gene generate oncogenic fusion proteins responsible for different myeloid diseases, including MDS/MPN and myeloid leukemias (*9*, *10*, *61*). Notably, because of the location of the HDAC3 gene in a genomic region frequently deleted in MDS/MPN, loss of function of this histone deacetylase was previously suggested as a potential contributor to this disease (*37*). Our identification that Nup358 regulates HDAC3 and that this nucleoporin is an important player in hematopoietic progenitor homeostasis and differentiation suggests that alterations in Nup358 function might contribute to the pathogenesis of hematological cancers by impairing the differentiation of early MyPs.

## MATERIALS AND METHODS

### Mice

*Nup358^fl/fl^* mice were obtained from J. Van Deursen. Animals were bred in a specific pathogen–free facility. All experiments were approved by the Institutional Animal Care and Use Committee of Sanford Burnham Prebys Medical Discovery Institute. All mice were 9 to 12 weeks old. To generate *Nup358^fl/fl^-CreER^T2^* mice, Nup358^fl/fl^ mice were crossed with *R26-CreER^T2^* mice (The Jackson Laboratory). All mice were used at 9 to 12 weeks of age.

### Murine cell collection

Mice were euthanized by cervical dislocation after isoflurane anesthesia. Tibia and femur bones of hindlimbs were collected, and bone marrow cells were isolated by flushing the bones with 1.2% fetal bovine serum (FBS) in phosphate-buffered saline (PBS) using a 27G needle and then passed through a 40-μm nylon mesh. Red blood cells were lysed as described, and viable cells were counted. Spleen and thymus were mechanically disaggregated and filtered through a 70-μm cell strainer, and red blood cells were removed. Blood was collected by retro-orbital bleeding or intracardiac puncture.

### Hematopoietic progenitor isolation and culture

Lin^−^ progenitors were isolated from bone marrow by negative selection using the EasySepTM Mouse Hematopoietic Progenitor Cell Isolation Kit (STEMCELL Technologies). Briefly, bone marrow cells were incubated first with rat serum and with biotinylated antibodies directed against non-HSCs and nonprogenitor cells [CD5, CD11b, CD19, CD45R/B220, Ly6G/C(Gr-1), and TER119] and then with streptavidin-coated magnetic particles. Labeled cells are captured using a magnet, and the Lin-negative cells were collected in the flow through. Purified cells were cultured at 0.2 × 10^6^ cells/ml in Dulbecco’s modified Eagle’s medium (DMEM), 15% heat inactivated FBS, SCF (20 ng/ml), IL-6 (10 ng/ml), and IL-3 (6 ng/ml) (Peprotech) for 24 hours and then treated with vehicle or 4-hydroxytamoxifen for 72 hours. For HDAC3 inhibition, RGFP966 (Selleckchem, S7229) was added to the cultures 48 hours after tamoxifen treatment at 10 μM concentration. Cells were counted daily and harvested after 48 hours of treatment with HDAC3 inhibitor for flow cytometry studies.

### Flow cytometry

Cells (2 × 10^6^) were stained with the Zombie NIR Fixable Viability Kit and incubated at 4°C for 30 min with fluorochrome-conjugated primary antibodies (1:200) in staining buffer [Hanks’ balanced salt solution (HBSS) and 1.2% FBS]. The cells were analyzed immediately by flow cytometry or fixed in 4% paraformaldehyde for later analysis. For MPP2 cell cycle analysis, surface marker–stained cells were fixed and permeabilized using Cytofix/Cytoperm buffer, washed with Perm/Wash buffer, and labeled with Hoechst 33342. Flow cytometry was performed with an LSRFortessa X-20 instrument with FACSDIVA software (BD Biosciences). Data were analyzed in FlowJo software v10.0.8r1.

### Colony forming assay

Freshly isolated bone marrow cells (1.8 × 10^5^) were seeded in 35-mm dishes using MethoCult 3234 (STEMCELL Technologies) supplemented with SCF (20 ng/ml), IL-6 (10 ng/ml), and IL-3 (5 ng/ml) (Peprotech). Dishes were incubated in 37°C in a humidified atmosphere for 7 days. For imaging, plates were fixed with 10% paraformaldehyde for 15 min at 4°C, stained with 0.25% Crystal Violet (Electron Microscopy Sciences), and washed with PBS.

### Bone marrow–derived macrophages

Bone marrow cells were plated in nontreated cell culture dishes in DMEM/F12 (Corning), 10% FBS, and 15% of L929-conditioned medium and grown in a humidified incubator at 5% CO_2_ and 37°C.After 3 days, fresh medium was added to the culture. After 7 days, macrophages were seeded in 96-well plates and treated with 4-hydroxytamoxifen (0.5 μM, Sigma-Aldrich) for 10 days. Cells were then stained with Hoechst 33342 (8.3 ng/ml) and propidium iodide (1 ng/ml) and imaged with Celigo Imaging Cytometer (Nexcelom Bioscience).

### Hematopoietic progenitor cell isolation and culture

Lin-negative hematopoietic progenitors were isolated from total bone marrow cells suspension by negative selection using that EasySepTM Mouse Hematopoietic Progenitor Cell Isolation Kit (STEMCELL Technologies). Briefly, bone marrow cells were incubated first with rat serum and with biotinylated antibodies directed against non-HSCs and nonprogenitor cells [CD5, CD11b, CD19, CD45R/B220, Ly6G/C(Gr-1), and TER119] and then with streptavidin-coated magnetic particles. Labeled cells are captured using a magnet, and the Lin-negative cells were collected in the flow through. Purified Lin-negative cells were cultured in six-well plates at 0.2 × 10^6^ cells/ml in DMEM, 15% heat inactivated FBS, SCF (20 ng/ml), IL-6 (10 ng/ml), and IL-3 (6 ng/ml) (Peprotech) for 24 hours at 37°C and subsequently treated with vehicle or 4-hydroxytamoxifen for 72 hours. Cells were counted daily. For HDAC3 inhibition, Lineage-negative progenitors were isolated and cultured. After 48 hours of tamoxifen treatment, the selective HDAC3 inhibitor RGFP966 (Selleckchem, S7229) was added to the cultures at a 10 μM concentration. For HDAC1 and HDAC2 inhibition, Lineage-negative progenitors were isolated and cultured. After 48 hours of tamoxifen treatment, the selective HDAC1 and HDAC2 inhibitor Romidepsin (Selleckchem, S3020) was added to the cultures at a 10 nM concentration. Viable cells were counted daily and harvested after 48 hours of treatment with different HDAC inhibitors for flow cytometry studies.

### Bone marrow transplantation

For bone marrow transplantation experiments, 2.5 million bone marrow cells isolated from *Nup358^fl/fl^* (CD45.2) and *Nup358^fl/fl^CreER^T2^* (CD45.2) were injected in the tail vein of recipient mice (CD45.1) previously lethally irradiated. After 11 weeks, transplanted mice were administered daily with 2 mg of tamoxifen by intraperitoneal injection for three consecutive days. After 5 days from the first tamoxifen treatment, hematological analysis was performed on peripheral blood collected by retro-orbital bleeding. Transplanted mice were euthanized after 12 days from the first tamoxifen injection for ethical reasons.

### siRNA transfection

293T cells were obtained from American Type Culture Collection (CRL-321) and cultured in humidified conditions at 37°C with 5% CO_2_ in DMEM with 10% heat-inactivated FBS (Sigma-Aldrich). Cells were transfected with scramble or Nup358-specific siRNAs (Horizon Discovery, D-001810-02-05 and J-004746-10-0010) using Lipofectamine RNAiMax and analyzed at 72 hours.

### Viral transduction

Virus was prepared by the Sanford Burnham Prebys Viral Vectors Core. MOLM13 and 293T cells were transduced with pTRIPZ tetracycline-inducible short hairpin RNA (shRNA) lentiviruses (control RHS4743 or Nup358 targeting 200761584, 200765221, and 200766435 from Dharmacon), followed by 12 days selection with Puromycin (1.25 μg/ml; Gibco). Expression of shRNA was induced with doxycycline (200 ng/ml; Clontech).

### Nup210 gene targeting using CRISPR-Cas9

Nup210 single guide RNAs (sgRNAs, Synthego) were transfected together with SpCas9 2NLS (Synthego) into 293T cells using Neon Transfection System (Invitrogen) according to the manufacturer’s instructions. The Nup210-targeting sgRNA sequences were: sgNup210 #1 UGCUGCUCGUCCAGGCCCAG and sgNup210 #2 CGAAGAUGAUGCUGGUGAGG. Negative control sgRNA (mod) #1 (Synthego) was used as nontargeting sgRNA. Nup210 CRISPR knockout mixed population was used for subsequent analysis.

### Immunofluorescence

Cells were fixed in 4% paraformaldehyde for 5 min at room temperature (RT). For the detection of Ubc9 at the NPCs, cells were fixed with cold methanol for 5 min at −20°C. Blocking was performed in immunofluorescence (IF) buffer [PBS, 1% bovine serum albumin (BSA), 0.02% SDS, and 0.1% Triton X-100] for 30 min at RT. Primary antibodies were incubated overnight at 4°C. Cells were washed three times with IF buffer, incubated with secondary antibodies, labeled with Hoechst 33342, and washed with PBS. Antibodies are listed as Supplementary Materials. Images were taken with a DMi8 Leica SP8 confocal microscope and analyzed in Leica Application Suite X software v3.1.5.16308. Relative intensity of nuclear and cytoplasmic HDAC3 was calculated using ImageJ v2.0.0-rc-54/1.51 h (NIH) by measuring the mean intensity fluorescence of regions of interest of the same area in the nucleoplasm and cytoplasm.

### HDAC3 coimmunoprecipitation

293T cells were harvested and washed twice with PBS and homogenized in immunoprecipitation (IP) buffer [50 mM Tris (pH 7.4), 150 mM NaCl, 0.5% NP-40, 1 mM EDTA, 1 mM MgCl_2_, and 1 mM dithiothreitol] containing HALT Protease and Phosphatase Inhibitor Cocktail (Thermo Fisher Scientific) and 20 mM *N*-Ethylmaleimide to inhibit deSUMOylation. DNA was sheared using a 29G syringe. Samples were centrifuged at 3000*g* at 4°C for 10 min to remove debris. Protein concentration was measured using the Pierce bicinchoninic acid (BCA) reagent. Eight milligrams of total proteins were diluted to the final concentration of 2 mg/ml in IP buffer containing protease and isopeptidase inhibitors and incubated with Protein A/G Magnetic Beads (Thermo Fisher Scientific) for 2 hours to remove nonspecific binding. Precleared protein solution was incubated overnight at 4°C on a rotator with either a specific anti-HDAC3 antibody (Cell Signaling Technology, 85057) at the final dilution of 1:400 or with the same amount of control rabbit IgG. The next morning, 80 μl of Protein A/G Magnetic Beads were added, and samples were incubated on a rotator for 4 hours at 4°C. After collection of the flow through, immunocomplexes were washed three times with IP buffer and eluted from the magnetic beads by incubation with NuPAGE LDS sample buffer (Thermo Fisher Scientific) with reducing agent for 15 min at 70°C and analyzed by immunoblot. For mass spectrometry analysis, beads containing affinity-purified proteins were resuspended with 8 M urea and 50 mM ammonium bicarbonate. Protein disulfide bonds were reduced with 5 mM tris(2-carboxyethyl) phosphine at 30°C for 60 min, and cysteines were subsequently alkylated with 15 mM iodoacetamide in the dark at RT for 30 min. Urea was then diluted to 1 M urea using 50 mM ABC, and proteins were subjected to overnight digestion with mass spec grade Trypsin/Lys-C mix (Promega). Following overnight digestion, beads were pulled down and the solution with peptides transferred to a new tube. Last, samples were acidified with formic acid (FA) and subsequently desalted using AssayMap C18 cartridges mounted on an Agilent AssayMap BRAVO liquid handling system. Cartridges were first conditioned with 100% acetonitrile (ACN) followed by 0.1% FA; samples were then loaded, washed with 0.1% FA, and peptides eluted with 60% ACN and 0.1% FA. The samples were dried down and resuspended in 2% ACN + 0.1 FA.

### LC-MS/MS analysis

Before liquid chromatography tandem mass spectrometry (LC-MS/MS) analysis, dried enriched peptides were reconstituted with 2% ACN and 0.1% FA, and concentration was determined using a NanoDrop spectrophometer (Thermo Fisher Scientific). Samples were then analyzed by LC-MS/MS using a Proxeon EASY-nanoLC system (Thermo Fisher Scientific) coupled to a Q-Exactive Plus mass spectrometer (Thermo Fisher Scientific). Peptides were separated using an analytical C18 Aurora column (75 μm by 250 mm, 1.6-μm particles; IonOpticks) at a flow rate of 200 nl/min using a 120-min gradient: 1 to 5% B in 1 min, 6 to 23% B in 72 min, 23 to 34% B in 45 min, and 34 to 48% B in 2 min (A = FA 0.1%; B = 80% ACN and 0.1% FA). The mass spectrometer was operated in positive data–dependent acquisition mode. MS1 spectra were measured in the Orbitrap in a mass-to-charge ratio (*m*/*z*) of 350 to 1700 with a resolution of 70,000 at *m*/*z* 400. Automatic gain control target was set to 1 × 10^6^ with a maximum injection time of 100 ms. Up to 12 MS2 spectra per duty cycle were triggered; fragmented by HCD; and acquired with a resolution of 17,500 and an AGC target of 5 × 10^4^, an isolation window of 1.6 *m*/*z*, and a normalized collision energy of 25. The dynamic exclusion was set to 20 s with a 10–part per million (ppm) mass tolerance around the precursor. All mass spectra were analyzed with MaxQuant software version 1.6.11.0. MS/MS spectra were searched against the *Homo sapiens* UniProt-reviewed protein sequence database (downloaded in January 2020) and GPM cRAP sequences (commonly known protein contaminants). Precursor mass tolerance was set to 20 and 4.5 ppm for the first search where initial mass recalibration was completed and for the main search, respectively. Product ions were searched with a mass tolerance 0.5 Da. The maximum precursor ion charge state used for searching was 7. Carbamidomethylation of cysteine was searched as a fixed modification, while oxidation of methionine and acetylation of protein N-terminal were searched as variable modifications. Enzyme was set to trypsin in a specific mode, and a maximum of two missed cleavages was allowed for searching. The target-decoy–based false discovery rate filter for spectrum and protein identification was set to 1%.

### Immunoblotting

Cells were homogenized with radioimmunoprecipitation assay (RIPA) buffer containing HALT Protease and Phosphatase Inhibitor Cocktail (Thermo Fisher Scientific). DNA was sheared using a 29G syringe. Samples were centrifuged at 3000*g* at 4°C for 10 min to remove debris. Protein concentration was determined using the Pierce BCA reagent. Thirty to eighty micrograms of protein were resolved by SDS–polyacrylamide gel electrophoresis (PAGE) on NuPAGE 3 to 8% Tris-acetate gels or NuPAGE 4 to 12% Bis-Tris gels (Life Technologies) and blotted to nitrocellulose membranes. Membranes were blocked with tris-buffered saline with 0.1% Tween 20 (TBS-T) containing 5% nonfat milk and incubated with primary antibodies diluted in TBS-T overnight at 4°C. Membranes were developed using SuperSignal West Pico Plus Chemiluminescent Substrate (Thermo Fisher Scientific).

### HDAC3 and SUMOylation inhibition

293T cells were exposed to vehicle or HDAC3 inhibitor RGFP966 (Selleckchem, S7229) at a final concentration 20 μM for 24 hours. To inhibit protein SUMOylation, 25 μM 2-D08 (Selleckchem, S8696) was used for 3 days (*62*).

### Antibodies used for flow cytometry

Antibodies used for flow cytometry are as follows: B220/CD45R (BD Biosciences, 561880), CD115 (BD Biosciences, 743642), CD11b (BioLegend, 101228), CD16/32 Fc Block (BioLegend, 101302), CD11c (BioLegend, 117330), CD135 (BioLegend, 135306), CD150 (BioLegend, 115909 and 115914), CD19 (BioLegend, 115540), CD3ε (BioLegend, 100336), CD4 (BioLegend, 100406), CD45 (eBioscience, 56-0451-80), CD48 (BioLegend, 103422), CD49b (BioLegend, 108907), CD8a (BD Biosciences, 563786), cKit (BioLegend, 105806), F4/80 (BioLegend,123133), Lineage cocktail (BioLegend, 79724), Ly-6C (BD Biosciences, 553104), Ly-6G (BioLegend, 127618), Sca1 (BioLegend, 108134), and Siglec-F (BD Biosciences 552126). Different cell populations were identified as follows: CLP (Lin^−^CD127^+^Sca1^lo^c-Kit^lo^CD135^hi^), LMPP (Lin^−^CD127^−^Sca1^hi^c-Kit^hi^CD135^hi^), MyPs, (Lin^−^CD127^−^Sca1^−^cKit^+^), monocytes (CD45^+^CD11b^+^Ly6C^+^), neutrophils (CD45^+^CD11b^+^Ly6G^+^), macrophages (CD45^+^CD11b^+^F4/80^+^), CD4^+^T cells (CD45^+^CD3^+^CD4^+^CD8^−^), CD8^+^T cells (CD45^+^CD3^+^CD8^+^CD4^−^), B cells (CD45^+^B220^+^CD3^–^), natural killer cells (CD45^+^CD3^−^CD49b^+^), B cells (CD45 + B220+), LSK cells (Lin^−^Sca1^+^cKit^+^), MPP2 (Lin^−^Sca1^+^cKit^+^ CD135^−^CD48^+^CD150^+^), MPP3 (Lin^−^Sca1^+^cKit^+^CD135^−^CD48^+^CD150^−^), MPP4 (Lin^−^Sca1^+^cKit^+^CD135^+^), HSC^LT^ (Lin^−^Sca1^+^cKit^+^CD135^−^CD48^−^CD150^+^), and HSC^ST^ (Lin^−^Sca1^+^cKit^+^CD135^−^CD48^−^CD150^−^).

### Antibodies used for immunofluorescence and immunoblotting

Antibodies used for immunofluorescence and immunoblotting are as follows: mAb414 (Covance, MMS-120P or BioLegend, 902902), anti-Nup358/RanBP2 (Bethyl Laboratories, A301-796A), anti-Nup98 (Cell Signaling Technology, 2598P), anti-HDAC3 (Cell Signaling Technology, 3949), anti-HDAC1 (Cell Signaling Technology, 34589), anti-HDAC2 (Cell Signaling Technology, 5118), anti-RB1 (R&D, MAB6495), anti-p53 (Cell Signaling Technology, 2524), anti-RBL2 (Sigma-Aldrich, HPA019703), anti-DMTF1 (Novus Biologicals, NBP1-84073), anti-E2F1 (Santa Cruz Biotechnology, sc-193), anti-PU.1/Spi1 (Abcam, ab88082), anti-GTF2I (Invitrogen, PA5-17642), anti-Ubc9 (Abcam, ab33044), anti-Nup358 (Santa Cruz Biotechnology, sc74518), anti-HDAC3 (Cell Signaling Technology, 3949), anti-Hsp90 (R&D Systems MAB3286), anti-Nup210 (Bethyl, A301-795A), anti-Nup96 (Bethyl, A301-784A), and anti-SUMO2/3 (Cell Signaling Technology, 4971).

### scRNA-seq data analysis

Bone marrow cells were stained for flow cytometry with Lin antibody cocktail and with antibodies against Sca1 and cKit as indicated in the “Flow cytometry” section. Before sorting, cells were resuspended in HBSS, 1% BSA, 20 mM Hepes (pH 7.2). Live Lin^−^Sca1^+^cKit^+^ cells were sorted at 4°C on a BD FACSAria II Cell Sorter (BD Biosciences) with a 70-μm nozzle into DMEM without glucose, glutamine, sodium pyruvate, and phenol red and supplemented with 10% FBS. Purity was verified after each sort and was found to be > 99%. Sorted LSK cells were loaded into a Chromium Next GEM Chip and processed with the Chromium Controller (10x Genomics) according to the manufacturer’s instructions for single-cell barcoding. Libraries were prepared using Chromium Next GEM Single Cell 3′ Reagent Kits v3.1 (10x Genomics) and were sequenced to an average depth of about 50,000 reads per cell. Data were analyzed by ROSALIND (https://rosalind.bio/), with a HyperScale architecture developed by ROSALIND Inc. Quality scores were assessed using FastQC. Cell Ranger was used to align reads to the *Mus musculus* genome build GRCm38, count UMIs, call cell barcodes, and perform default clustering. Individual sample reads were normalized via Relative Log Expression using DESeq2 R library. Read distribution percentages, violin plots, identity heatmaps, and sample MDS plots were generated as part of the QC step using RSeQC. DEseq2 was also used to calculate fold changes and *P* values and perform optional covariate correction. Clustering of genes for the final heatmap of differential expression was done using the partitioning around medoids method using the fpc R library. Hypergeometric distribution was used to analyze the enrichment of pathways, gene ontology, domain structure, and other ontologies. The topGO R library^,^ was used to determine local similarities and dependencies between GO terms to perform Elim pruning correction. Several database sources were referenced for enrichment analysis, including Interpro, NCBI, MSigDB, REACTOME, and WikiPathways. Enrichment was calculated relative to a set of background genes relevant for the experiment.
