# Supplementary material for: Nucleoporin Nup358 drives the differentiation of myeloid-biased multipotent progenitors by modulating HDAC3 nuclear translocation
Source: Sci Adv. 2024 Jun 5;10(23):eadn8963. doi: 10.1126/sciadv.adn8963 (PMC11152124; doi:10.1126/sciadv.adn8963)
Supplement: Supplementary file 1 — Figs. S1 to S9 Legends for tables S1 to S5 [file sciadv.adn8963_sm.pdf]

Science Advances
AAAS

Supplementary Materials for

Nucleoporin Nup358 drives the differentiation of myeloid-biased multipotent progenitors by modulating HDAC3 nuclear translocation

Valeria Guglielmi *et al.*

Corresponding author: Maximiliano A. D'Angelo, mdangelo@sbpdiscovery.org

Sci. Adv. **10**, eadn8963 (2024)
DOI: 10.1126/sciadv.adn8963

The PDF file includes:

Figs. S1 to S9
Legends for tables S1 to S5

Other Supplementary Material for this manuscript includes the following:

Tables S1 to S5

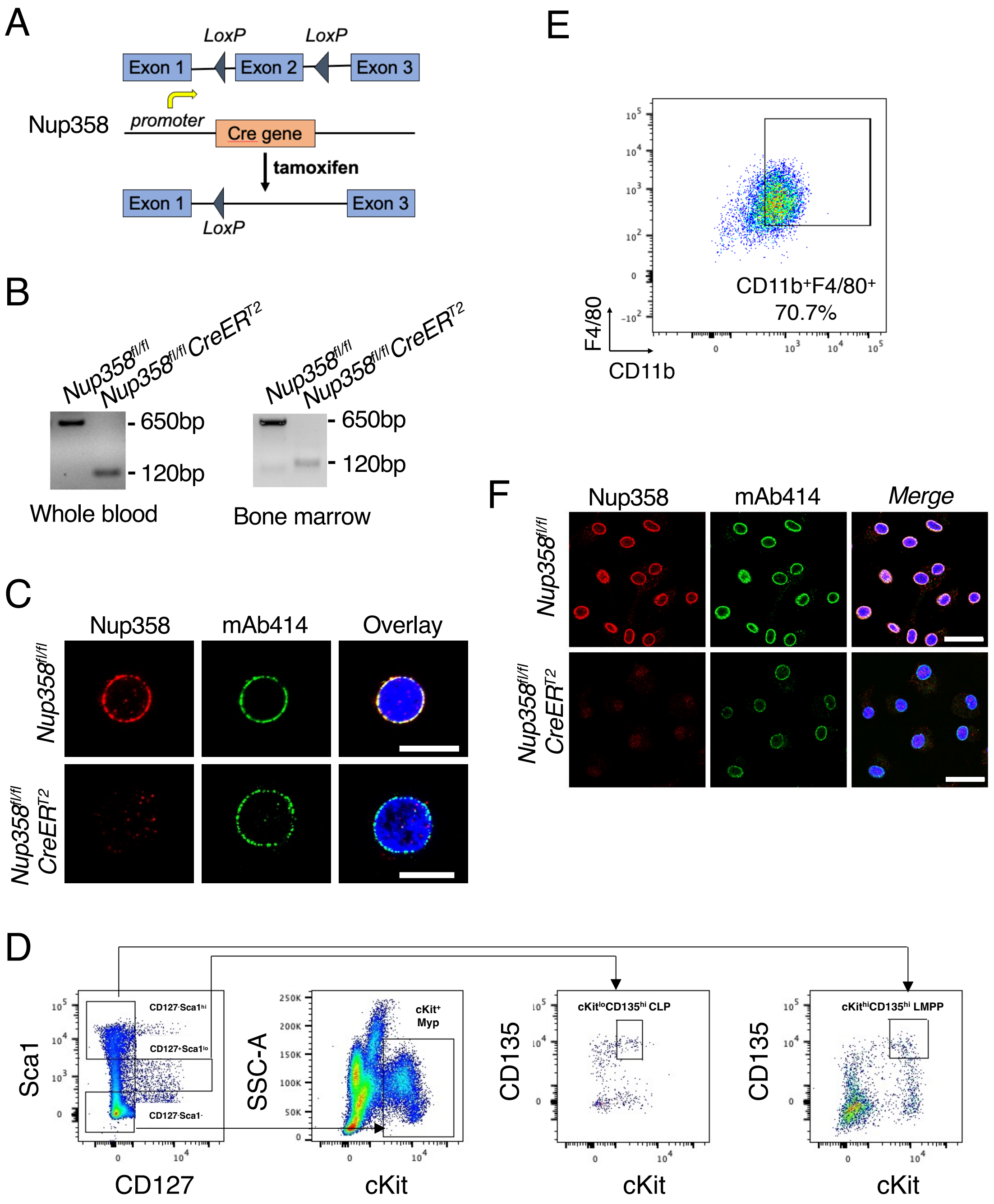

Fig. S1. Nup358 knockout strategy. (A) Schematic illustration of Nup358 knockout strategy. (B) PCR genotyping of whole blood and bone marrow cells after tamoxifen treatment showing the effective recombination of exon 2 in *Nup358$^{fl/fl}$CreER^{T2}* mice. (C) Immunofluorescence analysis of Nup358 and the NPC marker mAb414 that recognizes several nucleoporins on hematopoietic progenitors isolated from control and Nup358 knockout mice. Cells were co-stained with Hoechst. Scale bars, 10 µm. (D) Gating strategy used to identify common myeloid progenitors (CMP), lymphoid-primed multipotent progenitors (LMPP), and common lymphoid progenitors (CLP). (E)

Flow cytometry analysis of *in vitro* differentiated macrophages. (F) Bone marrow cells were isolated from *Nup358*$^{fl/fl}$ and *Nup358*$^{fl/fl}$*CreER*^{T2} mice and differentiated into macrophages. Differentiated cells were treated with tamoxifen and stained with an antibody against Nup358 and the mAb414 NPC marker. Scale bars, 25 μm.

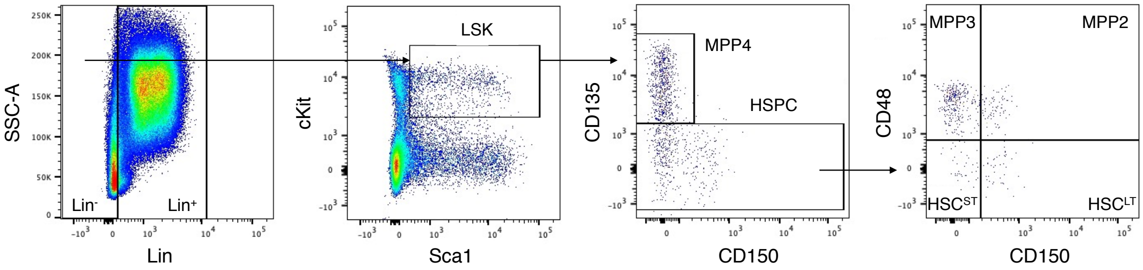

Fig. S2. Gating strategy used to identify multipotent progenitors LSK, HSC^{LT}, HSCST, MPP2, MPP3, and MPP4 cells.

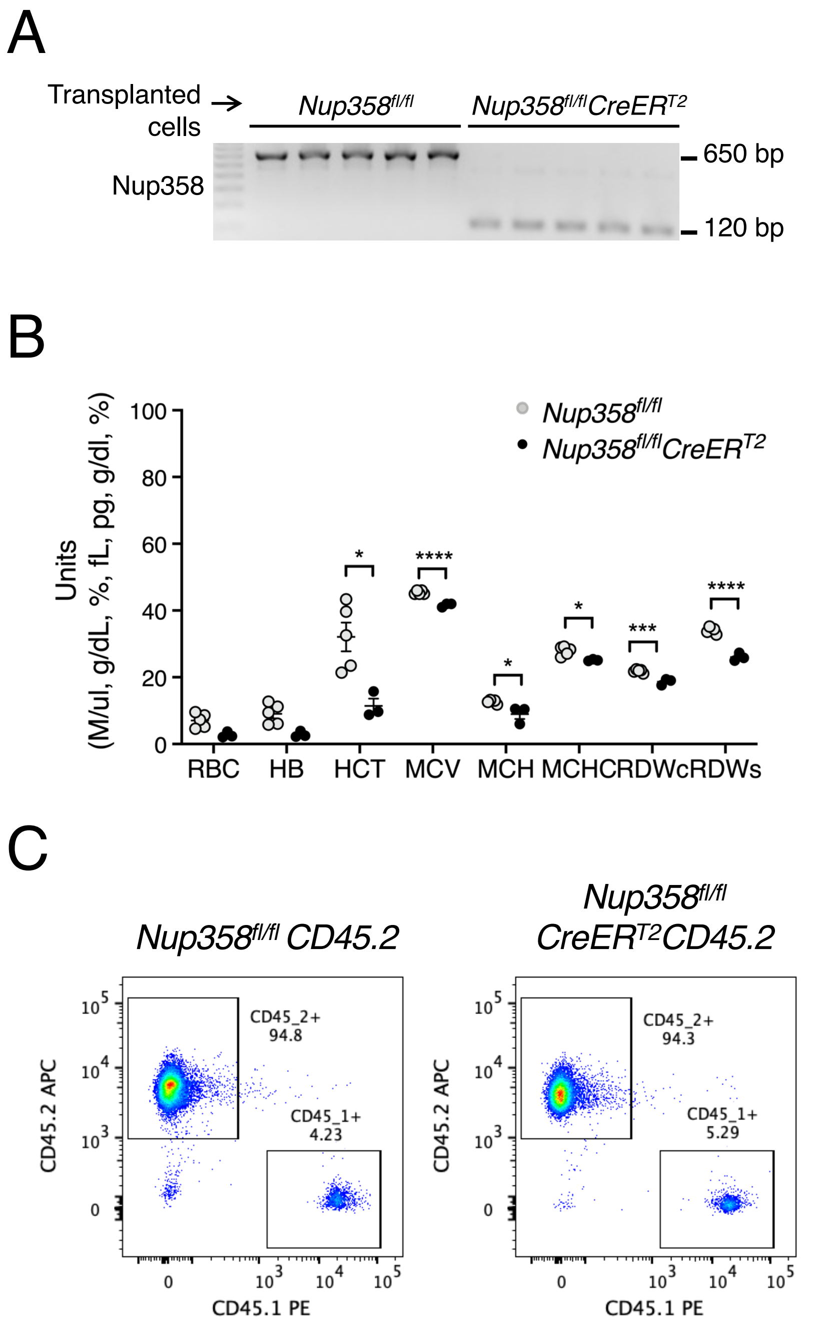

Fig. S3. Transplantation assays. (A) PCR genotyping showing recombination of Nup358 exon 2 in peripheral blood of CD45.1 wild type mice transplanted with *Nup358*$^{fl/fl}$*CreER*^{T2} cells after 12 weeks of reconstitution and 5 days from the first tamoxifen administration. (B) Hematological analysis of peripheral blood of CD45.1 wild type mice transplanted with either *Nup358*$^{fl/fl}$ or *Nup358*$^{fl/fl}$*CreER*^{T2} after 12 weeks of reconstitution and 5 days from the first tamoxifen administration showing the decrease in hematocrit (HCT), mean corpuscular volume (MCV), mean corpuscular hemoglobin (MCH), mean corpuscular hemoglobin concentration (MCHC), and

red cell distribution width (RCDs and RDCs). (C) Flow cytometry analysis of the expression of the congenic markers CD45.1 and CD45.2 in peripheral blood of CD45.1 wild type mice transplanted with either *Nup358*$^{fl/fl}$CD45.2 or *Nup358*$^{fl/fl}$*CreER*^{T2}CD45.2 after 12 weeks of reconstitution and 12 days from the first tamoxifen administration. Data are mean ± s.d. * $p \leq 0.05$, ** $p \leq 0.01$, ***≤ 0.001, ****≤ 0.0001 by multiple unpaired Student's t test with Holm-Sidak method to correct for multiple comparisons. Each dot represents an individual animal.

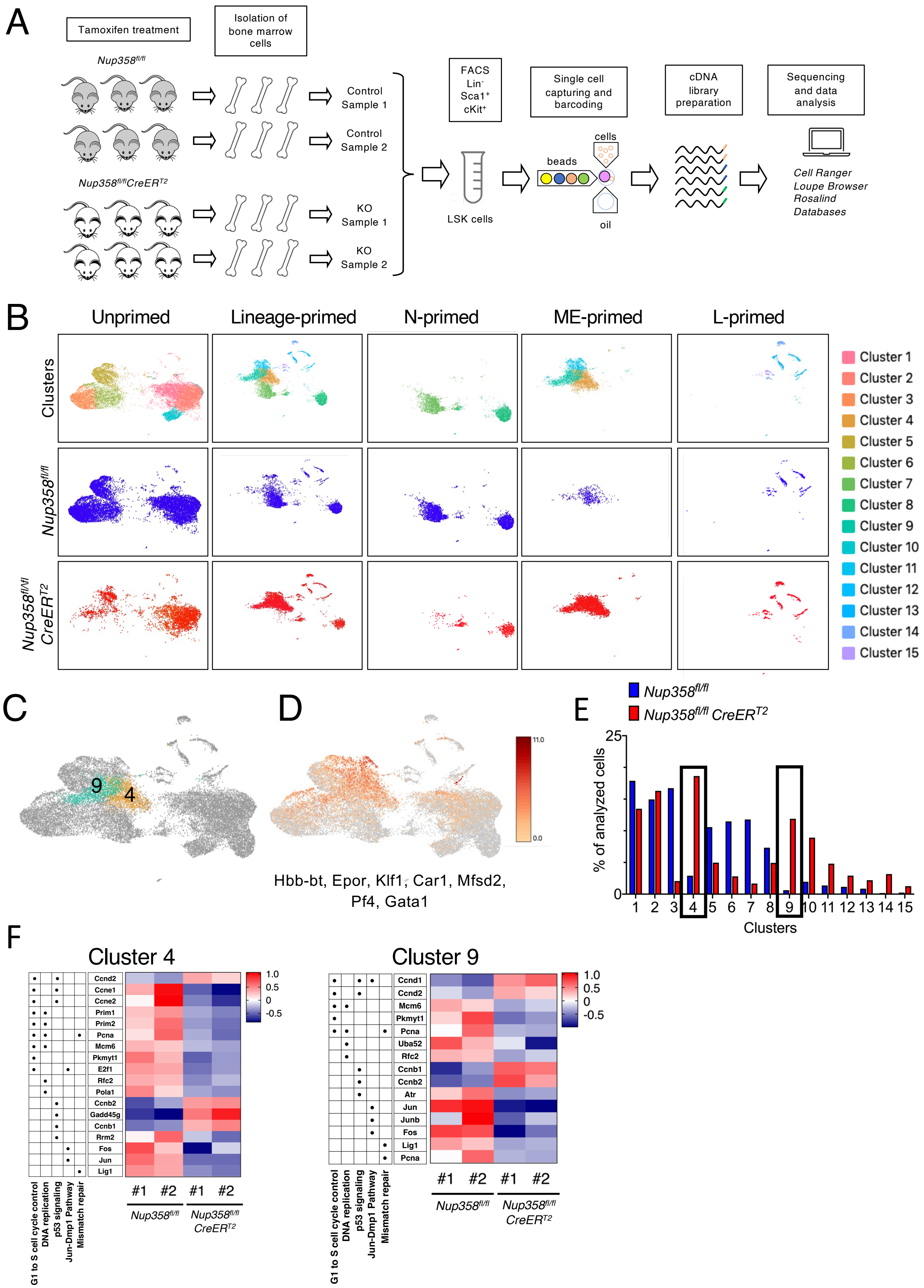

A
Tamoxifen treatment
Isolation of bone marrow cells
Nup358fl/fl
Control Sample 1
Control Sample 2
Nup358fl/flCreERT2
KO Sample 1
KO Sample 2
FACS Lin- Sca1+ cKit+
LSK cells
Single cell capturing and barcoding
beads
cells
oil
cDNA library preparation
Sequencing and data analysis
Cell Ranger
Loupe Browser
Rosalind
Databases
B
Unprimed
Lineage-primed
N-primed
ME-primed
L-primed
Clusters
Nup358fl/fl
Nup358fl/fl CreERT2
Cluster 1
Cluster 2
Cluster 3
Cluster 4
Cluster 5
Cluster 6
Cluster 7
Cluster 8
Cluster 9
Cluster 10
Cluster 11
Cluster 12
Cluster 13
Cluster 14
Cluster 15
C
9
4
D
11.0
0.0
Hbb-bt, Epor, Klf1, Car1, Mfsd2, Pf4, Gata1
E
Nup358fl/fl
Nup358fl/fl CreERT2
% of analyzed cells
25
0
1 2 3 4 5 6 7 8 9 10 11 12 13 14 15
Clusters
F
Cluster 4
Ccnd2
Ccne1
Ccne2
Prim1
Prim2
Pcna
Mcm6
Pkmyt1
E2f1
Rfc2
Pola1
Ccnb2
Gadd45g
Ccnb1
Rrm2
Fos
Jun
Lig1
1.0
0.5
0
-0.5
#1 #2 #1 #2
Nup358fl/fl
Nup358fl/fl CreERT2
G1 to S cell cycle control
DNA replication
p53 signaling
Jun-Dmp1 Pathway
Mismatch repair
Cluster 9
Ccnd1
Ccnd2
Mcm6
Pkmyt1
Pcna
Uba52
Rfc2
Ccnb1
Ccnb2
Atr
Jun
Junb
Fos
Lig1
Pcna
1.0
0.5
0
-0.5
#1 #2 #1 #2
Nup358fl/fl
Nup358fl/fl CreERT2
G1 to S cell cycle control
DNA replication
p53 signaling
Jun-Dmp1 Pathway
Mismatch repair

Fig. S4. Single cell RNAseq approach and analysis. (A) Schematic illustration of scRNA-seq experimental strategy. (B) UMAP representation of cluster differences between control and Nup358 knockout mice. (C) UMAP representation megakaryocyte-erythrocyte primed clusters (4 and 9). (D) Expression of megakaryocyte/erythrocyte genes superimposed to the UMAP plot. Combined expression level of the megakaryocyte/erythrocyte genes Hbb-bt, Epor, Klf1, Car1, Mfsd2, Pf4, and Gata1 is represented in shades of red/orange. (E) Percentage of control (blue) and Nup358 knockout (red) cells in each cluster. Megakaryocyte-erythrocyte primed clusters (4 and 9) are highlighted in boxes. (F) Heatmaps of differentially expressed genes involved in cell cycle and DNA damage pathways in cluster 4 and 9 (Fold change >+/- 1.25; $p<0.05$).

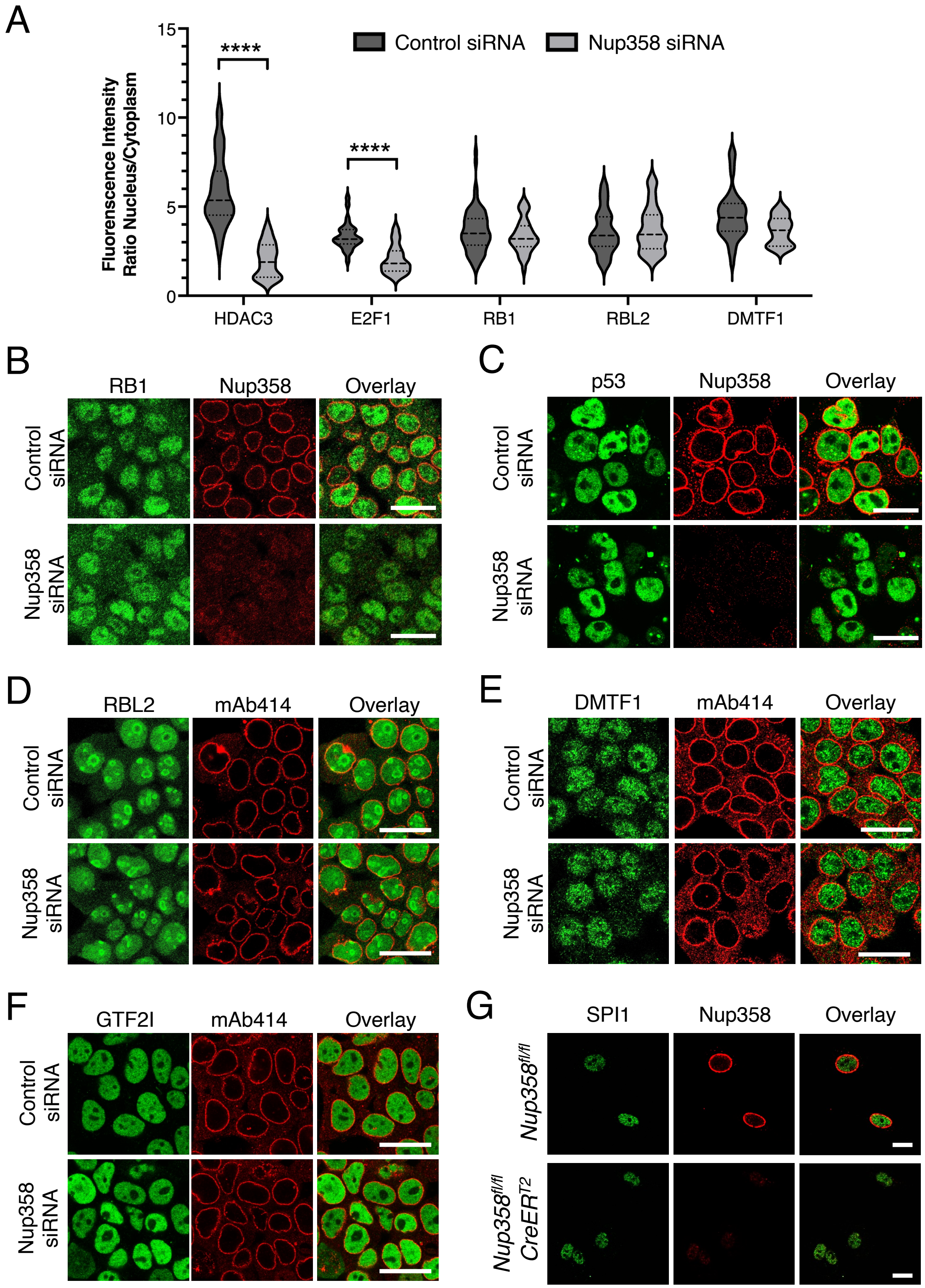

Fig. S5. Effect of Nup358 depletion on the localization of transcriptional regulators. 293T cells were transfected with scramble control or Nup358 specific siRNAs and stained with antibodies against HDAC3, E2F1, RB1, RBL2, and DMTF1 at 72 hours post-transfection. (A) The

ratio between nuclear and cytoplasmic signal for each factor was quantified. (B-F) Representative immunofluorescence images showing the localization of RB1 (B), p53 (C), RBL2 (D), DMTF1 (E), and GTF2I (F) in 293T cells 72 hours after transfection with scramble control or Nup358 specific siRNAs. Cells were co-stained with an antibody against nucleoporin Nup358 or with the mAb414 antibody that recognizes several nucleoporins. Scale bars, 25 µm. (G) Bone marrow derived macrophages were obtained from *Nup358*$^{fl/fl}$ and *Nup358*$^{fl/fl}$*CreER*^{T2} and treated *in vitro* with tamoxifen to ablate Nup358 expression. The localization of SPI1 (PU.1) was analyzed by immunofluorescence. Scale bars, 25 µm.

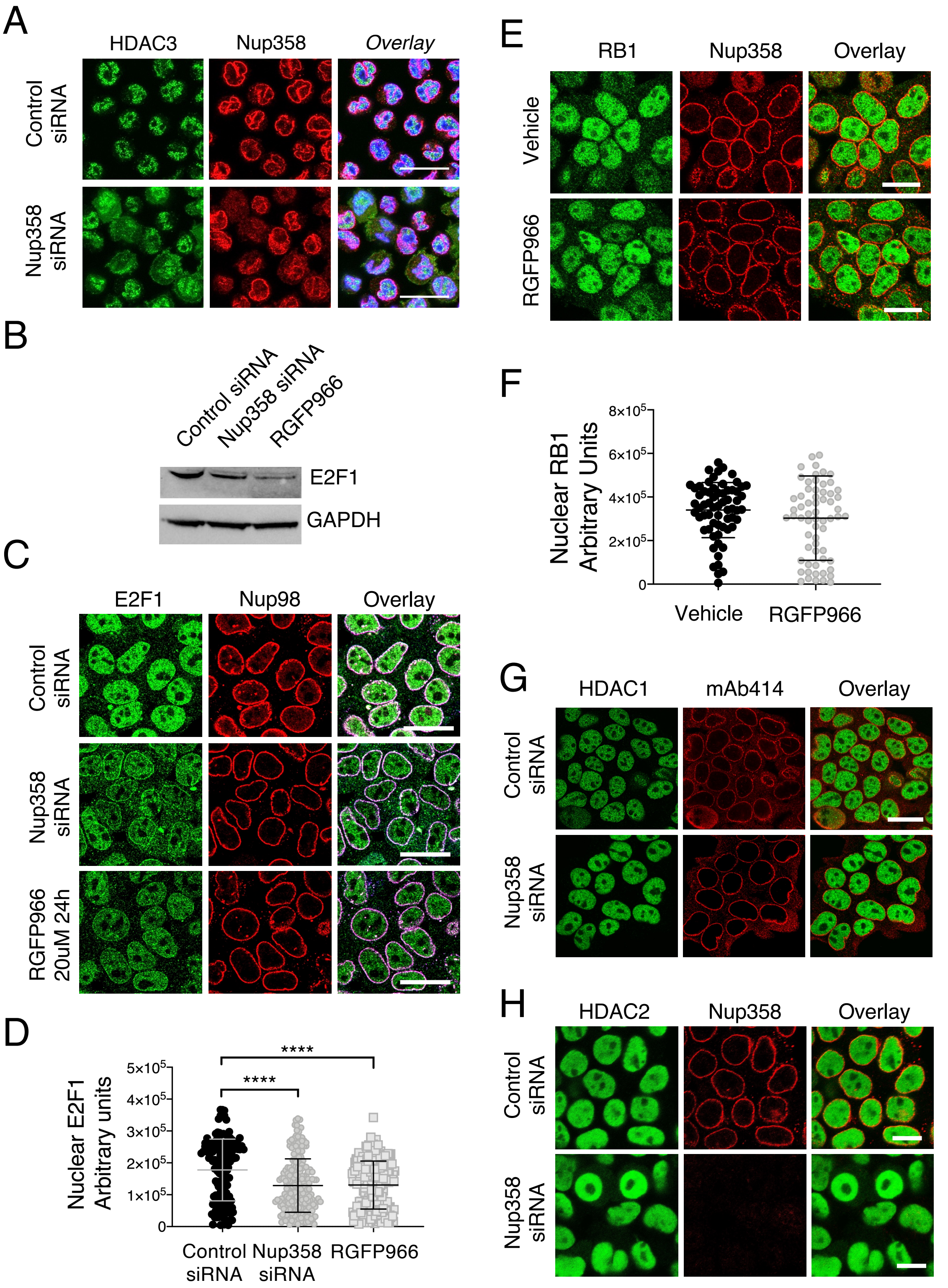

Fig. S6. Nup358 depletion E2F1, HDAC1, and HDAC2. (A) MOLM13 cells were transduced with Lentivirus carrying either scramble control or Nup358 targeting shRNA and the localization

of HDAC3 was analyzed by immunofluorescence after 21 days of treatment with doxycycline to induce downregulation of Nup358. (B) 293T cells were transfected with scramble control or Nup358-specific siRNAs or treated with the selective HDAC3 inhibitor RGFP966 at the final concentration of 20 μM for 24 hours and the expression levels of E2F1 were analyzed by western blot. GAPDH was used as loading control. (C) 293T cells were transfected with scramble control or Nup358-specific siRNAs or treated with the selective HDAC3 inhibitor RGFP966 at the final concentration of 20 μM for 24 hours and the localization of E2F1 was analyzed by immunofluorescence. Cells were co-stained with an antibody against nucleoporin Nup98. Scale bars, 25 μm. (D) Quantification of E2F1 nuclear signal in control and Nup358 knockdown 293T cells and 293T cells treated with RGFP966 as in (C). (E) 293T cells were treated with the selective HDAC3 inhibitor RGFP966 at the final concentration of 20 μM for 24 hours and the localization of RB1 was analyzed by immunofluorescence. Cells were co-stained with an antibody against nucleoporin Nup358. Scale bars, 25 μm. (H) Quantification of RB1 nuclear signal in 293T cells after treatment with RGFP966 as in (G). (G,H) 293T cells were transfected with scramble control or Nup358 specific siRNAs and the localization of HDAC1 (G), and HDAC2 (H) was analyzed by immunofluorescence at 72 hours post-transfection. Cells were co-stained with either the NPC marker mAb414 that recognizes several nucleoporins or an antibody against nucleoporin Nup358. Scale bars, 25 μm.

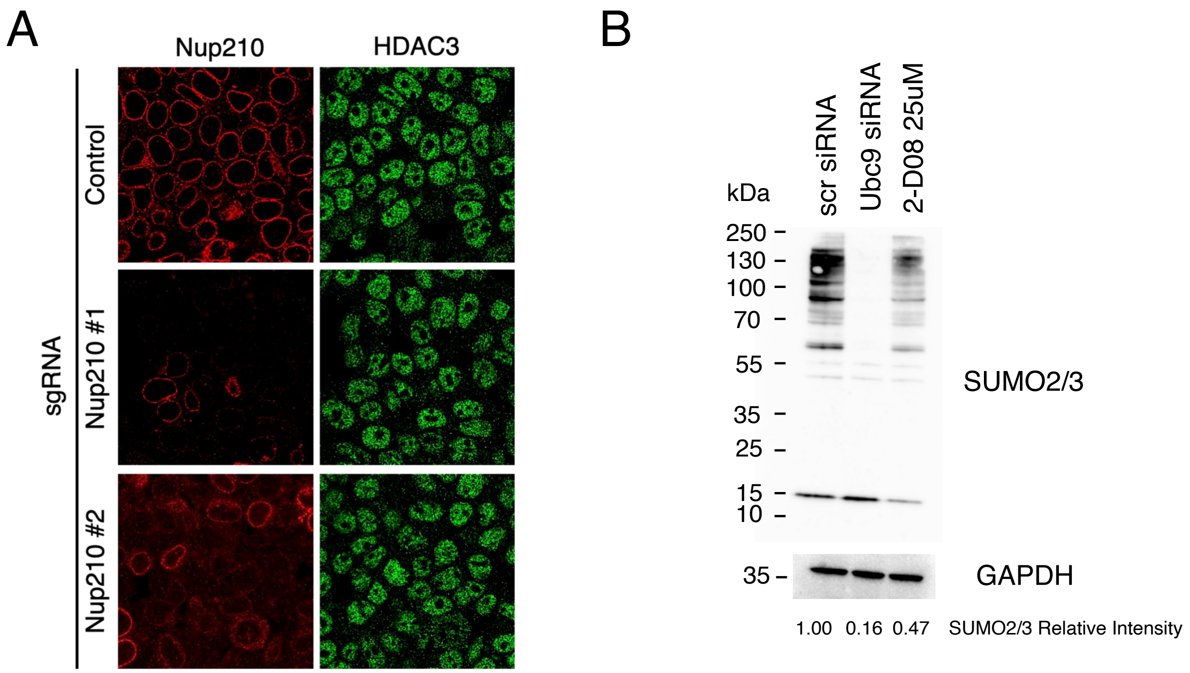

Fig. S7. **Nup210 depletion effect on HDAC3 and SUMOylation inhibition controls.** (A) 293T cells were transfected with control or Nup210 sgRNAs together with SpCas9 2NLS protein. Nup210 CRISPR knockout mixed populations were analyzed antibodies against HDAC3 or Nup210. Ablation of Nup210 does not affect HDAC3 localization. Scale bars, 50 µm. (B) 293T cells were transfected with scramble control or Nup358 specific siRNAs or treated with the SUMOylation inhibitor 2-DO8 for 72 hours at 25 µM. Inhibition of SUMOylation upon Ubc9 downregulation or 2-DO8 treatment was verified by immunoblot analysis on total protein extracts using an antibody against SUMO2/3.

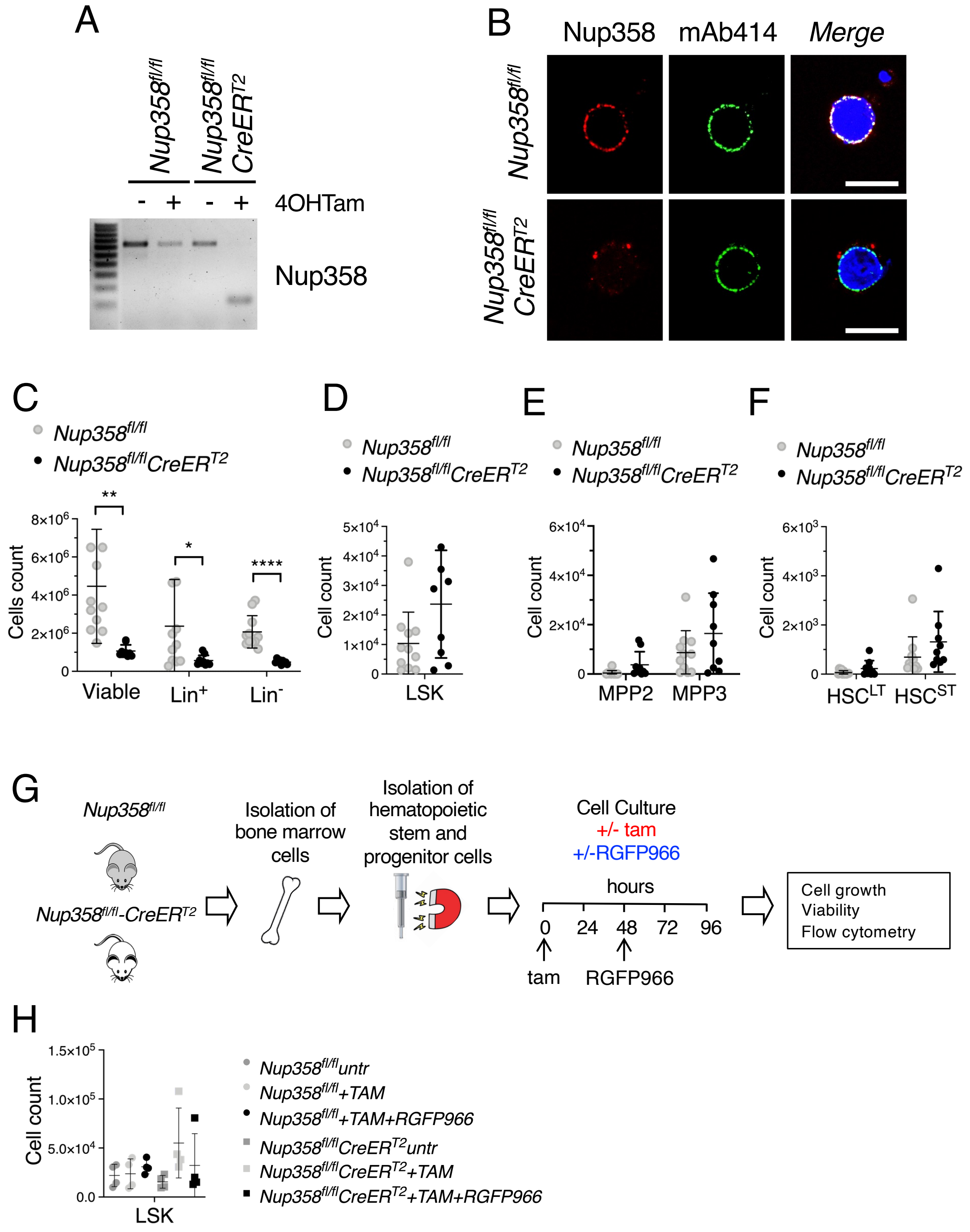

Fig. S8. *In vitro* myeloid differentiation system. (A) PCR genotyping after 96 hours of tamoxifen treatment confirming the elimination of exon 2 from Nup358 in hematopoietic progenitor cultures. (B), Immunofluorescence analysis of Nup358 and NPCs (mAb414) on hematopoietic progenitors isolated from Nup358$^{fl/fl}$ and *Nup358$^{fl/fl}$CreER^{T2}* mice and treated in vitro with tamoxifen for 96 hours. Scale bars, 10 µm. (C-F) Flow cytometry analysis of hematopoietic progenitor cell cultures 96 hours after tamoxifen treatment. (G) Schematic illustration of experimental strategy to evaluate the effect of HDAC3 inhibition in control and Nup358 knockout hematopoietic progenitors.

Hematopoietic progenitors isolated from Nup358$^{fl/fl}$ or *Nup358$^{fl/fl}$CreER^{T2}* mice were cultured in vitro in conditions that promote myeloid differentiation in presence of tamoxifen alone or in combination with the selective HDAC3 inhibitor RGFP966. (H) Number of LSK cells determined by flow cytometry analysis of hematopoietic progenitor cultures treated with either tamoxifen or vehicle for 96 hours and HDAC3 inhibitor RGFP966 or vehicle for 48 hours.

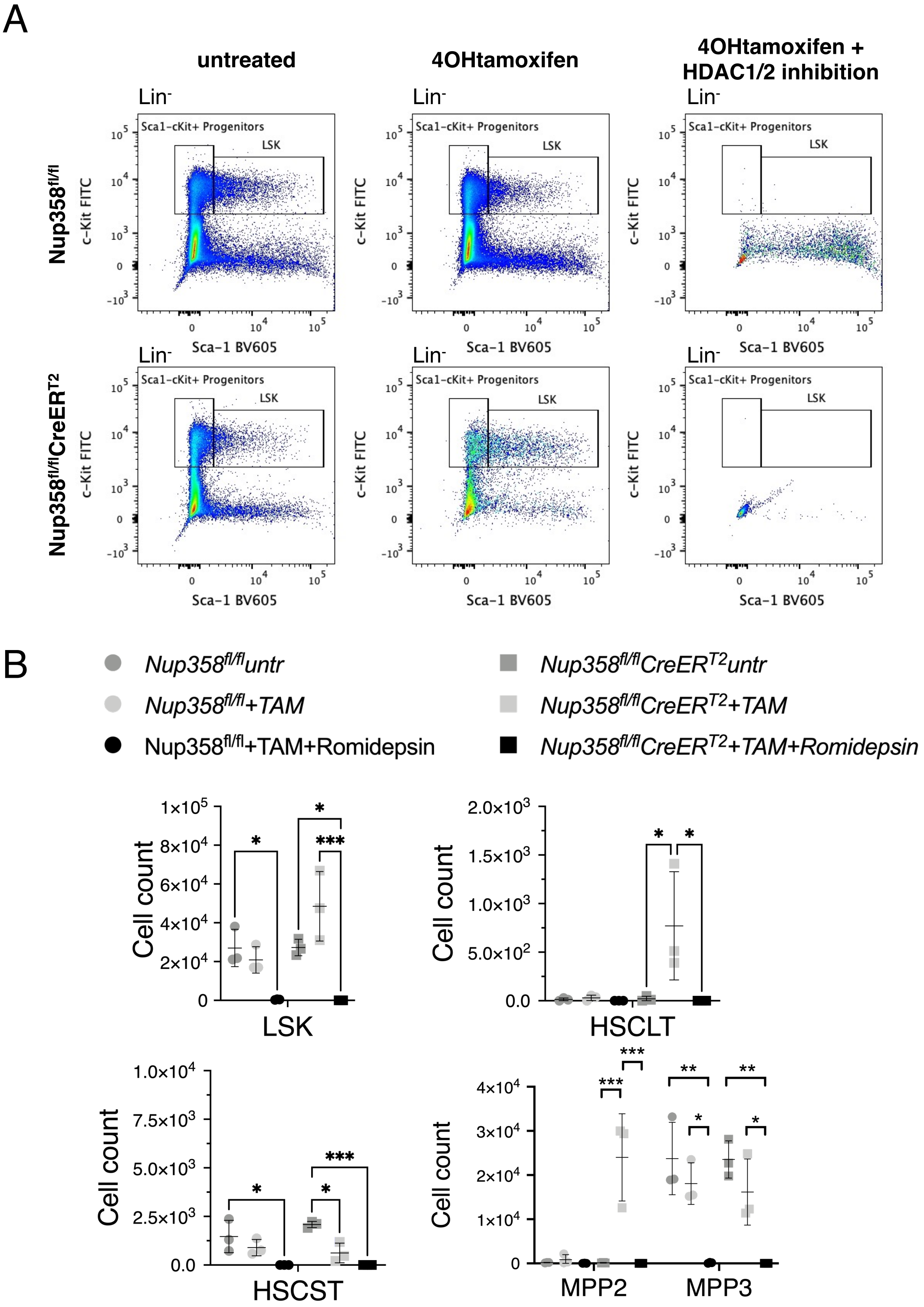

Fig. S9. Effect of HDAC1 and HDAC2 inhibition in control and Nup358 knockout hematopoietic progenitors. Hematopoietic progenitors isolated from *Nup358$^{fl/fl}$* or *Nup358$^{fl/fl}$CreER^{T2}* mice were cultured *in vitro* in conditions that promote myeloid differentiation

in presence of tamoxifen alone or in combination with the dual HDAC1/ HDAC2 inhibitor Romidepsin at the final concentration of 10 nM. (A) Flow cytometry analysis shows that treatment with Romidepsin eliminates $Lin^{-}Sca1^{-}cKit^{+}$ and LSK cells in both *Nup358*$^{fl/fl}$ and *Nup358*$^{fl/fl}$*CreER*^{T2} hematopoietic progenitor cultures. (B) Number of LSK and LSK subpopulations MPP2, MPP3, HSC^{ST}, and HSC^{LT} determined by flow cytometry analysis of hematopoietic progenitor cultures treated with either tamoxifen or vehicle for 96 hours and dual HDAC1/ HDAC2 inhibitor Romidepsin for 48 hours at the final concentration of 10 nM. LSK and LSK subpopulation MPP2, MPP3, HSC^{ST}, and HSC^{LT} are eliminated upon treatment with Romidepsin in both *Nup358*$^{fl/fl}$ and *Nup358*$^{fl/fl}$*CreER*^{T2} hematopoietic progenitor cultures.

Data S1. (separate file)

Supplemental Table 1. scRNAseq Sample Quality Control
Supplemental Table 2. Gene markers of the 15 clusters
Supplemental Table 3. Markers from Literature used for Cluster Annotation
Supplemental Table 4. Cluster 4 and 9-Differentally expressed genes ctr vs Nup358 KO
Supplemental Table 5. Proteomic analyses of HDAC3 interactors
